# Diversity and Pathogenicity of Six *Diaporthe* Species from *Juglans regia* in China

**DOI:** 10.3390/jof10080583

**Published:** 2024-08-16

**Authors:** Aoli Jia, Lu Lin, Yixuan Li, Xinlei Fan

**Affiliations:** 1 The Key Laboratory of Efficient Production of Forest Resources, Beijing Forestry University, Beijing 100083, China; nice2cu@bjfu.edu.cn (A.J.); lulin0677@bjfu.edu.cn (L.L.); yixuanli@bjfu.edu.cn (Y.L.); 2 The Key Laboratory for Silviculture and Conservation of the Ministry of Education, Beijing Forestry University, Beijing 100083, China

**Keywords:** Diaporthales, pathogens, phylogeny, taxonomy

## Abstract

Walnut (*Juglans regia* L.) is cultivated extensively in China for its substantial economic potential as a woody oil species. However, many diseases caused by *Diaporthe* greatly affect the health of *Juglans regia* trees. The present study revealed the presence of *Diaporthe* species from *Juglans regia*. A total of six species of *Diaporthe* were isolated from twigs of *Juglans regia* in three provinces in China, including two known species (*Diaporthe gammata* and *D. tibetensis*) and four novel species (*D. chaotianensis*, *D. olivacea*, *D. shangluoensis* and *D. shangrilaensis*). Phylogenetic relationships of the new species were determined by multilocus phylogenetic analyses based on partial sequences of the internal transcribed spacer (ITS) region, calmodulin (*cal*) gene, histone H3 (*his3*) gene, translation elongation factor 1-α (*tef1-α*) gene and β-tubulin (*tub2*) gene. Pathogenicity tests indicated that all *Diaporthe* species obtained in this study were confirmed as pathogens of *Juglans regia*. This study deepens the understanding of species associated with several disease symptoms in *Juglans regia* and provides useful information for effective disease control.

## 1. Introduction

The walnut tree (*Juglans regia* L.), a perennial deciduous species, stands out as an economically significant hardwood tree cultivated worldwide for its nutritious nuts and valuable timber. Leading in global production are the United States and China, accounting for 30% (611,280 tons) and 43% (0.88 million tons) of the total fruit production worldwide, respectively [1]. Walnut seeds are also a high-density source of nutrients, particularly rich in proteins and essential fatty acids. The production of walnut seeds has increased rapidly worldwide in recent years. However, various diseases affect the condition of walnuts, thereby diminishing their economic potential. For example, twelve species of genera *Botryosphaeria*, *Diaporthe*, *Diplodia*, *Dothiorella*, *Lasiodiplodia* and *Neofusicoccum* cause cankers and blights of *Juglans regia* [2]; species of genera *Colletotrichum* and *Fusarium* cause serious leaf-spot disease [3,4]; and species of genera *Diaporthe* and *Neofusicoccum* can also cause fruit blight disease [5].

The genus *Diaporthe* (syn. *Phomopsis*) was established by Nitschke (1870) [6] with *D. eres* as the type species. Species of this genus are distributed global world with a wide host range and can occur as plant pathogens, endophytes and saprobes [7,8,9,10,11,12,13,14,15,16,17]. Over 1200 species epithets of *Diaporthe* have been recorded in the Index Fungorum (https://indexfungorum.org/; accessed on 1 July 2024). The sexual morph of *Diaporthe* generally has immersed ascomata and erumpent pseudostroma with elongated perithecial necks. Asci are unitunicate and sessile producing hyaline ascospores [7]. The asexual morph of *Diaporthe* can be identified by ostiolate conidiomata, cylindrical phialides and three types (alpha, beta and gamma) of conidia. All three types of conidia are aseptate and hyaline, while alpha conidia are fusiform, usually biguttulate; beta conidia are filiform, straight or more often hamate, and lack guttules; and gamma conidia are fusiform to subcylindrical, and multiguttulate [7,10].

The identification of *Diaporthe* has traditionally relied mainly on host associations and morphological characteristics such as the shape and size of ascomata, asci, ascospores, conidiomata, conidia and conidiophores [7,18,19,20,21]. The initial concept of *Diaporthe* species was founded on the premise of host specificity [19], which has given rise to the designation of nearly 2000 species names for *Diaporthe* and *Phomopsis*. Nonetheless, the validity of identifying species within this genus based solely on host associations and morphological features is contentious. Previous studies have shown that the morphological characters of many *Diaporthe* species are not always stable, as they may vary with the environment [10,18,22]. Recent studies demonstrated that most *Diaporthe* species could be found on diverse hosts and could co-occur on the same host or lesion with different life patterns [20,21,23,24]. Therefore, identification and description of species based on host association and morphological characters are not reliable within *Diaporthe* [10,25,26]. Currently, a polyphasic taxonomic approach combining phylogenetic and morphological analyses is widely employed [10,13,17,25,26,27,28]. Five genetic sequences (ITS, *cal*, *his3*, *tef1-α* and *tub2*) are widely used for phylogenetic analyses [27,28,29].

To date, a total of 26 *Diaporthe* species have been recorded to infect *Juglans regia* trees (https://nt.ars-grin.gov/fungaldatabases, accessed on 1 July 2024), of which eight are found in China: *D. amygdali*, *D. biguttulata*, *D. dejiangensis, D. eres*, *D. juglandigena, D. shennongjiaensis*, *D. tibetensis* and *D. tongrensis* [12,30,31,32,33,34]. Among these species, only *D. amygdali* has been confirmed as a causal agent of twig canker disease in *Juglans regia* [30]. Consequently, there is a clear necessity for additional research into the diversity and pathogenicity of *Diaporthe* species isolated from *Juglans regia* in China.

**Figure 1:**
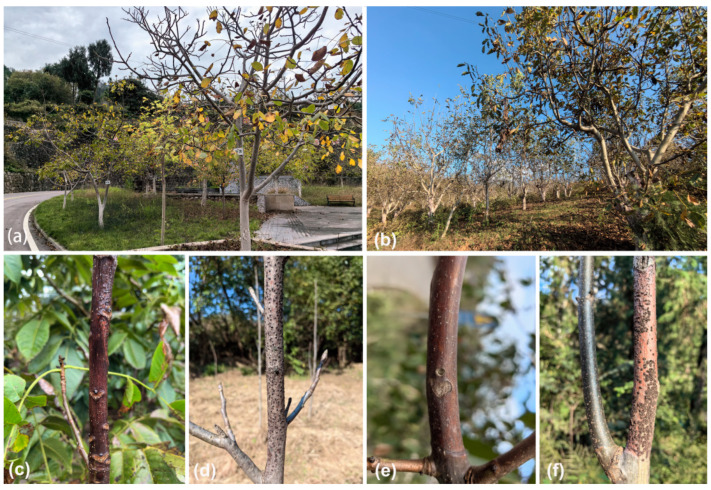
Canker and dieback diseases caused by *Diaporthe* species in *Juglans regia*. (**a**,**b**) Disease of the *Juglans regia* caused by *Diaporthe* in the field. (**c**–**f**) The trees infected by *Diaporthe*.

During the investigation of pathogens causing tree cankers or dieback diseases in Shaanxi, Sichuan and Yunnan provinces of China, branches with typical canker symptoms were collected and subsequently identified combining modern taxonomic concepts (Figure 1). Therefore, the objectives of the present study were to (i) identify *Diaporthe* taxa associated with dieback diseases of *Juglans regia* collected in this study and (ii) test the pathogenicity of species collected on *Juglans regia*.

## 2. Materials and Methods

### 2.1. Sampling and Isolation

During the survey conducted in 2022 and 2023, 122 diseased (branches and twigs with canker symptoms) *Juglans regia* branch samples were collected from five *Juglans regia* plantations in Shaanxi, Sichuan and Yunnan in China. Approximately 15–25 *Juglans regia* trees were sampled from each site, and cankered tissues were collected from a single branch of each tree showing symptoms typical of branch canker and dieback for detailed examination and fungal isolation. In total, 45 samples were collected. A total of 31 *Diaporthe* isolates were obtained from 45 specimens by removing a mucoid spore mass from conidiomata and/or ascomata, spreading the suspension over the surface with potato dextrose agar (PDA) (200 g potatoes, 20 g glucose and 20 g agar/L water) in a Petri dish and incubating at 25 °C for up to 24 h. Hyphal tips were removed to a new PDA plate twice to obtain a pure culture. Specimens were deposited in the Museum of Beijing Forestry University (BJFC). Axenic cultures are maintained in the China Forestry Culture Collection Centre (CFCC).

### 2.2. Morphological Analyses

Species identification was based on morphological characteristics of the ascomata or conidiomata formed on infected host materials. Macromorphological features (structure and size of conidiomata, ascomata, ectostromatic disc and ostioles) were photographed using a Leica stereomicroscope (M205 FA) (Leica Microsystems, Wetzlar, Germany). Micromorphological features (conidiophores, conidiogenous cells, asci and conidia/ascospores) were photographed using a Nikon Eclipse 80i microscope (Nikon Corporation, Tokyo, Japan), equipped with a Nikon digital sight DS-Ri2 high-resolution colour camera with differential interference contrast. Over 20 conidiomata were sectioned and 50 conidia were selected randomly to measure their lengths and widths. Colony diameters were measured and the colony colours described after 3 days and 14 days according to the colour charts of Rayner (1970) [35].

### 2.3. DNA Extraction, PCR Amplification and Sequencing

Mycelium used for DNA extraction was grown on PDA for three days and obtained from the cellophane surface by scraping. The genomic DNA was extracted from axenic cultures using the modified CTAB method [36]. Sequences were amplified by PCR from the ITS, *cal*, *his3*, *tef1-α* and *tub2* genetic regions. The PCR mixtures for all genes included 10 μL Mix (Promega, Madison, MI, USA), 7 μL double deionized water, 1 μL of each primer and 1 μL template DNA. All primers and PCR conditions are listed in Table 1. PCR products were electrophoresed in 1% agarose gel, and the DNA was sequenced by the Sino Geno Max Biotechnology Company Limited (Beijing, China). DNA sequences generated by the forward and reverse primers combination were used to obtain consensus sequences using Seqman v. 7.1.0 (DNASTAR Inc., Madison, WI, USA).

### 2.4. Phylogenetic Analyses

The sequences obtained from this study were analyzed with the NCBIs GenBank nucleotide datasets. Alignments based on ITS, *cal*, *his3*, *tef1-α* and *tub2* sequence data, including sequences obtained from this study and those downloaded from GenBank (Supplementary Table S1), were first aligned using MAFFT v. 6 [37] and edited manually using MEGA v. 6.0 [38]. *Diaporthella corylina* (CBS 121124) was used as the outgroup in polygenic *Diaporthe* analyses. Phylogenetic analyses were performed with PhyML v. 3.0 for the maximum likelihood (ML) method [39] and MrBayes v. 3.1.2 for the Bayesian inference (BI) method [40].

The best-fit evolutionary models for each partitioned locus were estimated by MrModeltest v. 2.3 following the Akaike information criterion (AIC) in ML and BI analyses [41]. For ML analysis, RAxML-NG was used [42]. Bootstrap supports were estimated with 100 pseudoreplicates and the appropriate models for each gene. BI analyses were completed using a Markov chain Monte Carlo (MCMC) algorithm with Bayesian posterior probabilities (BPPs) [43]. Trees were sampled every 100th generation after two MCMC chains were run from random trees for 10 million generations, which stopped when the average standard deviation of split frequencies fell below 0.01. For the burn-in phase of each analysis, the first 25% of the trees were discarded and the remaining trees were assessed to calculate BPP [43]. FigTree v. 1.3.1 [44] was used to show phylograms. The sequence data of 31 isolates were deposited in GenBank; their accession numbers, together with those of the other species used for the analysis, are listed in Table S1. The multilocus sequence alignment was deposited in TreeBASE (www.treebase.org, accessed on 1 July 2024; study ID 31531). 

### 2.5. Pathogenicity Test

Three representative isolates from each identified *Diaporthe* species were selected for pathogenicity testing in this study (*D. chaotianensis*: CFCC 70718–70720; *D. gammata*: CFCC 70722–70724; *D. olivacea*: CFCC 70713, 70715, 70716; *D. shangluoensis*: CFCC 70728, 70729, 70731; *D. shangrilaensis*: CFCC 70703, 70705, 70706; *D. tibetensis*: CFCC 70702, 70710, 70711). The pathogenicity tests were conducted on 2–year–old *Juglans regia* trees 1.3 m high and 1.5 cm thick which were planted in the field at a nursery of the Forest Protection Lab (Beijing, China). After the leaves grew, they were inoculated under natural conditions to determine pathogenicity during early April 2024 (mean air temperature = 15 °C). Sterilized 5 mm diameter inoculation rings were used to scald the bark surface of each branch to a depth of 2 mm. Agar plugs of the same size were removed from 6-day-old colonies of selected isolates, inserted into the wounds, sealed with moistened cotton wool and protected with parafilm. Six replications were made for each isolate. One plant per isolate was used as the negative control, and an equal number of plants inoculated with PDA agar plugs without colonies were used as the positive control. After one week, the parafilm and cotton wool were removed. These inoculated plants were maintained in the field. Fourteen days after inoculation, the lengths of the spots on the bark surface were measured from the inoculation point upwards and downwards using a digital calliper and then averaged. All spots from the experimental and control groups were reisolated to verify that the morphological characteristics and DNA sequences were consistent with the original isolates, thus fulfilling the Koch hypothesis. Differences in lesion length between isolates were analyzed by one-way analysis of variance (ANOVA) followed by least significant difference (LSD) tests. Statistical analysis was carried out by SPSS v. 20.0 and considered as significant at *p* < 0.05.

**Table 1:** Genes used in this study with PCR primers, primer DNA sequence, optimal annealing temperature and corresponding references.

Locus	PCR Primers	PCR: Thermal Cycles: (Annealing Temp. in Bold)	References of Primers Used
ITS	ITS1	(95 °C: 30 s, 51 °C: 30 s, 72 °C: 1 min) × 35 cycles	[45]
	ITS4		
*cal*	CAL228F	(95 °C: 15 s, 54 °C: 20 s, 72 °C: 1 min) × 35 cycles	[46]
	CAL737R		
*his3*	CYLH3F	(95 °C: 30 s, 58 °C: 30 s, 72 °C: 1 min) × 35 cycles	[47,48]
	H3-1b		
*tef1-α*	728F	(95 °C: 15 s, 55 °C: 20 s, 72 °C: 1 min) × 35 cycles	[46]
	1567R		
*tub2*	T1	(95 °C: 30 s, 55 °C: 30 s, 72 °C: 1min) × 35 cycles	[48]
	Bt2b		

## 3. Results

### 3.1. Phylogeny

**Figure 2:**
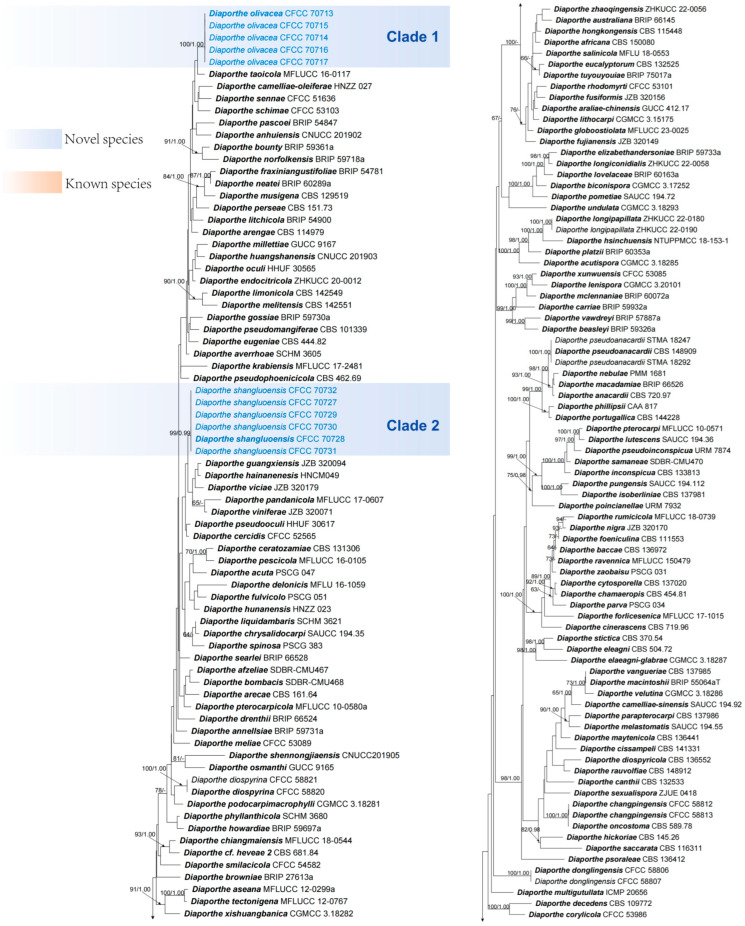

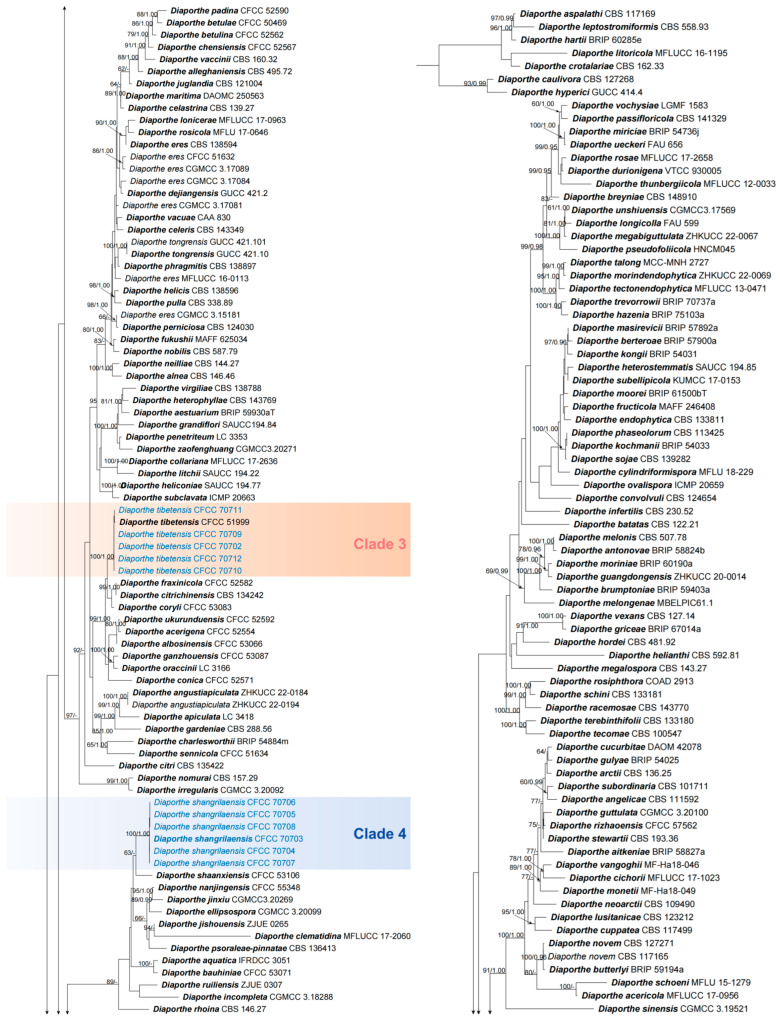

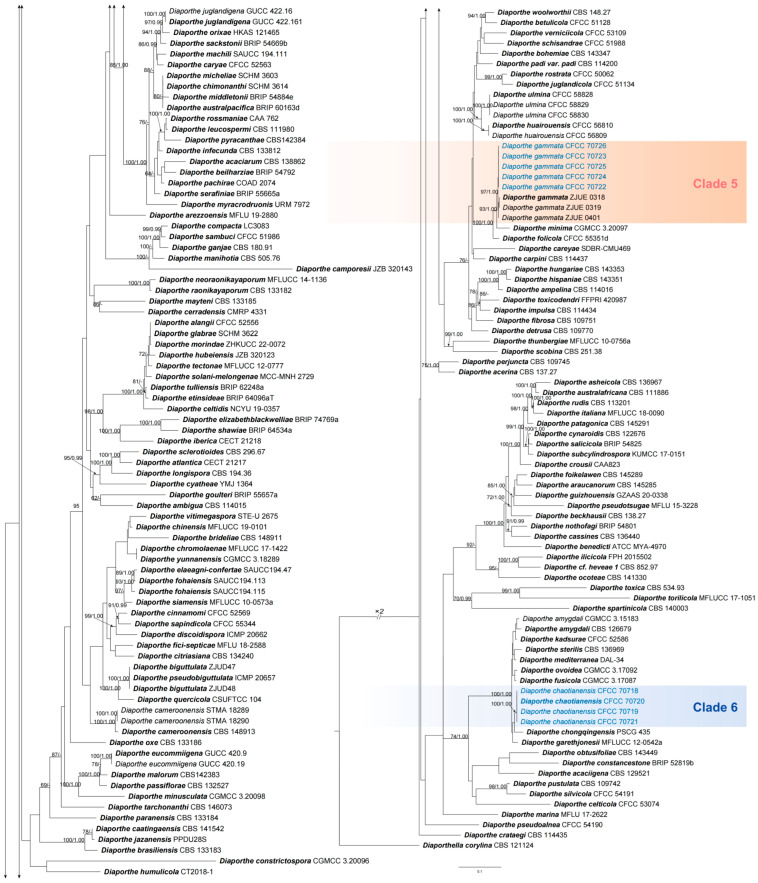
Phylogenetic tree of *Diaporthe* based on multiple gene loci (ITS, *cal*, *his3*, *tef1-α* and *tub2*) derived from ML analysis. ML bootstrap support values above 60% and Bayesian posterior probabilities above 0.95 are shown near nodes. Ex-type isolates are in bold. Strains in the current study are in blue. Isolates in this study are highlighted in two different colours. Clade 1–2, 4, 6 represent novel species. Clade 3, 5 represent known species.

Each gene region and the concatenated sequences of five genetic regions (ITS, *cal*, *his3*, *tef1-α* and *tub2*) were analyzed to infer the interspecific relationships within *Diaporthe*. The topological structures derived from each gene region were found to be consistent with those of the combined dataset (Figure 2, Figures S1–S5). The combined sequences dataset comprised 481 isolates (480 ingroup taxa including 31 new isolates in this study and one outgroup taxa, *Diaporthella corylina* CBS 121124). The sequence fragments were 3954 characters including gaps (548 for ITS, 952 for *cal*, 663 for *his3*, 785 for *tef1-α* and 1006 for *tub2*). The topologies resulting from ML and BI analyses of the concatenated dataset were similar. ML bootstraps (ML BS ≥ 60%) and Bayesian posterior probabilities (BPP ≥ 0.95) have been shown above the branches (Figure 2). For ML analysis, the matrix had 3044 distinct alignment patterns. The model parameters were as follows: A = 0.217335, C = 0.286382, G = 0.258070, T = 0.218908: substitution rates: AC = 0.866066, AG = 3.129585, AT = 0.966855, CG = 0.698073, CT = 3.794326, GT = 1.000000; gamma distribution shape parameter α = 0.538403; and likelihood value of ln: −124,733.462770.

The current 31 isolates clustered in six clades representing six species. Two represented known species (*D. gammata* and *D. tibetensis*) and four new clades. Isolates in clades 1, 2, 4 and 6 were separated from all other species and were also highly supported (ML/BI = 100/1) (Figure 2), representing four novel species (*D. chaotianensis*, *D. olivacea*, *D. shangluoensis* and *D. shangrilaensis*), which are detailed in the following sections.

### 3.2. Taxonomy

**Figure 3:**
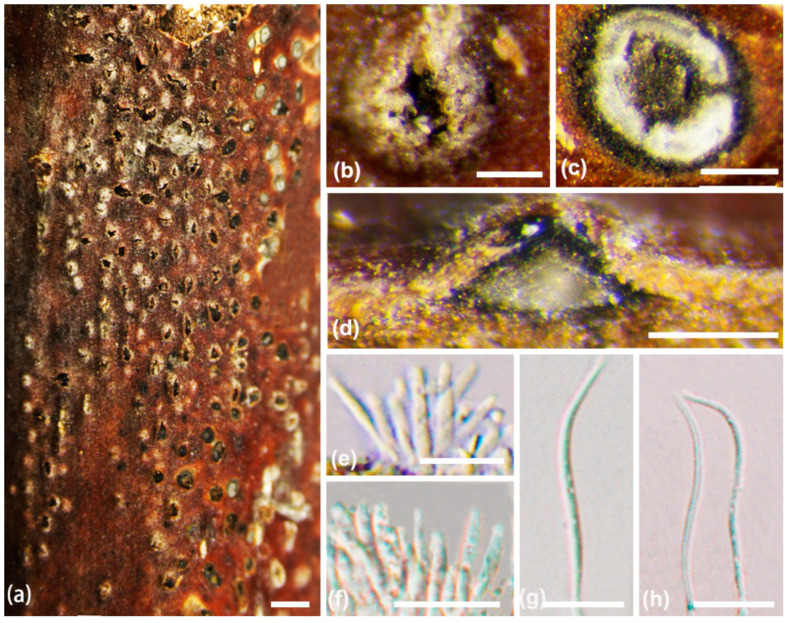
*Diaporthe chaotianensis* (BJFC-S2345). (**a**–**c**) Habit of conidiomata on twig; (**d**) longitudinal section through a conidioma; (**e**,**f**) conidiophores and conidiogenous cell; (**g**,**h**) beta conidia. Scale bars: 2 mm (**a**); 500 µm (**b**,**c**); 200 µm (**d**); 10 µm (**e**–**h**).

***Diaporthe chaotianensis*** A.L. Jia and X.L. Fan, sp. nov. (Figure 3)

*MycoBank*: MB 854191

*Etymology*: Named after the place where the fungus was isolated, Chaotian District, Guangyuan City.

*Typification*: China, Sichuan Province, Guangyuan City: Chaotian District, Zhongzi Town, 32°41′14″ N, 106°02′23″ E, from branches of *Juglans regia*, 10 October 2023, Y.X. Li, L. Lin and X.L. Fan (holotype BJFC-S2345, ex-holotype living culture CFCC 70720); 32°41′20″ N, 106°02′42″ E (paratype BJFC-S2346, ex-paratype living culture CFCC 70718).

*Description*: Sexual morph not observed. Asexual morph: conidiomata pycnidial, conical, immersed in bark, scattered, erumpent through the surface, with a solitary locule. Ectostromatic disc black to white, with one ostiole per disc, ovoid to circular. Locule undivided, 185–670 μm (av. = 370 μm, n = 20) diam. Conidiophores cylindrical, attenuate towards the apex, hyaline, phialidic, unbranched, slightly curved, 8–15 × 1–1.2 μm (av. = 11 ± 1.8 × 1.6 ± 0.3 μm, n = 50). Conidiogenous cells phialidic, hyaline, cylindrical, tapered towards the apex, 6–19 × 1–2 μm (av. = 10 ± 3.3 × 1.4 ± 0.3 µm, n = 50). Beta conidia hyaline, aseptate, filiform, straight or curved, tapering towards both ends, 21–32 × 1–2 μm (av. = 26 ± 3 × 1.4 ± 0.3 µm, n = 50). Alpha conidia are not observed.

**Figure 4:**
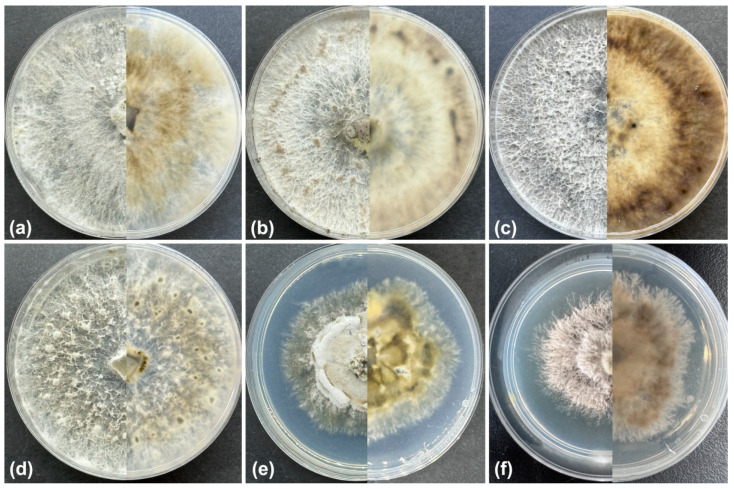
Colonies of *Diaporthe* species on potato dextrose agar (PDA). (**a**) *D. chaotianensis*; (**b**) *D. gammata*; (**c**) *D. olivacea*; (**d**) *D. shangluoensis*; (**e**) *D. shangrilaensis*; (**f**) *D. tibetensis*. (**a**–**f**) Colonies on potato dextrose agar after 7 days (**left**) and 10 days (**right**).

*Cultural characteristics*: Colonies initially white, grown to 63 mm after 3 days, compact at the centre and sparse at the surroundings, becoming honey after 7 days. Colonies are flat with a uniform texture, lacking aerial mycelium. Conidiomata were sparse, black, distributed irregularly (Figure 4a).

*Additional materials examined*: China, Sichuan Province, Guangyuan City: Chaotian District, Zhongzi Town, 32°41′21″ N, 106°02′25″ E, from branches of *Juglans regia,* 10 October 2023, Y.X. Li, L. Lin and X.L. Fan (BJFC-S2347, living culture CFCC 70719, 70721).

*Notes*: Four isolates represent the *D. chaotianensis* cluster in a clade distinct from other species of *Diaporthe* known from DNA sequence data. Phylogenetically, *D. chaotianensis* is most closely related to *D. chongqingensis* in phylogenetic trees of the combined dataset and *tef1-α* loci (Figure 2, Figure S4), but can be distinguished from *D. chongqingensis* based on phylogeny in ITS, *cal*, *his3* and *tub2* loci (15/506 for ITS, 3/558 for *cal*, 1/600 for *his3* and 11/641 for *tub2*). In the phylogenetic tree of ITS loci, *D. chaotianensis* forms an independent lineage closely related to *D. fusicola* and *D. kadsurae* (Figure S1), but can be distinguished from these species based on alignments of the separate loci (differs from *D. fusicola* in: 5/523 for ITS, 6/600 for *cal*, 11/677 for *tef1-α* and 5/632 for *tub2*; *D. kadsurae*: 5/523 for ITS, 3/483 for *his3*, 11/677 for *tef1-α* and 6/857 for *tub2*). In the phylogenetic trees of *cal* and *his3* loci, *D. chaotianensis* is closely related to *D. amygdali*, *D. mediterranea* and *D. sterilis* (Figures S2 and S3), but can be distinguished from these species based on alignments of the separate loci (differs from *D. amygdali* in: 11/523 for ITS, 2/600 for *cal*, 3/483 for *his3*, 13/677 for *tef1-α* and 15/935 for *tub2; D. mediterranea*: 10/523 for ITS, 3/600 for *cal*, 3/483 for *his3*, 11/677 for *tef1-α* and 7/935 for *tub2*; *D. sterilis*: 10/523 for ITS, 3/600 for *cal*, 19/483 for *his3*, 11/677 for *tef1-α* and 6/857 for *tub2*). In the phylogenetic tree of *tub2* loci, *D. chaotianensis* is closely related to *D. garethjonesii* (Figure S5), but can be distinguished from *D. chongqingensis* based on phylogeny in ITS, *cal*, *tef1-α* and *tub2* loci (11/523 for ITS, 8/653 for *cal*, 10/677 for *tef1-α* and 2/631 for *tub2*). Morphologically, *D. chaotianensis* differs from these species based on morphology. Conidiophores of *D. chaotianensis* are longer than *D. chongqingensis* (8–15 × 1–1.2 μm vs. 6.5–12.5 × 2–6 μm) [16], but smaller than *D. fusicola* (8–15 × 1–1.2 μm vs. 11–24.1 × 1.6–2.9 μm) [49] and narrower than *D. kadsurae* (8–15 × 1–1.2 μm vs. 7–11 × 1.8–2.9 μm) [12]. Conidiogenous cells of *D. chaotianensis* are smaller than *D. chongqingensis* (6–19 × 1–2 μm vs. 14–26 × 1.5–2.5 μm) [16]. Furthermore, *D. chaotianensis* differs from *D. amygdali*, *D. fusicola*, *D. kadsurae* and *D. mediterranea* in the production of beta conidia, which is not observed in these species [12,49,50]. *Diaporthe chaotianensis* differs from *D. garethjonesii* in its smaller conidia (21–32 × 1–2 μm vs. 40–50 × 3–4 μm) [51], and mainly differs from *D. sterilis* in its capacity to produce beta conidia, because all isolates representing *D. sterilis* could not be induced to sporulate on any of the culture media used by Lombard et al. [52], when this new *Diaporthe* species collected from *Vaccinium corymbosum* was described [52].

**Figure 5:**
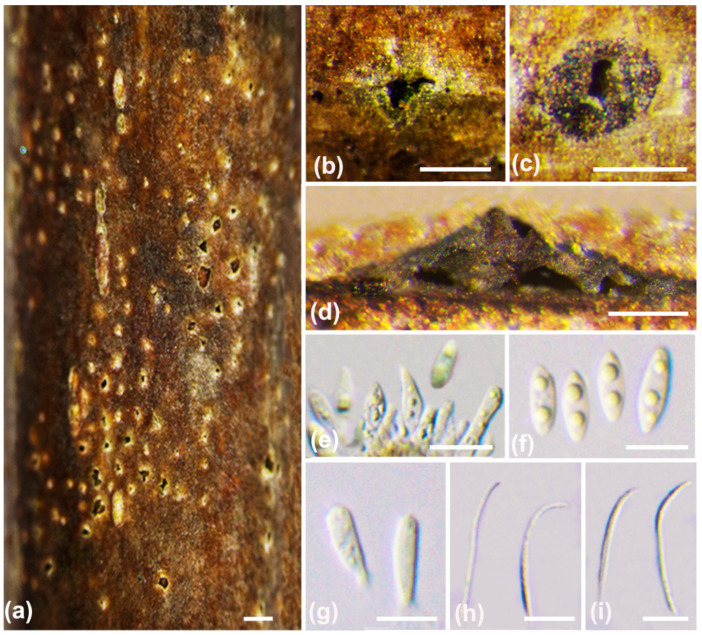
*Diaporthe gammata* (BJFC-S2348). (**a**–**c**) Habit of conidiomata on twig; (**d**) longitudinal section through a conidioma; (**e**) conidiophores and conidiogenous cell; (**f**) alpha conidia; (**g**) gamma conidia; (**h**,**i**) beta conidia. Scale bars: 2 mm (**a**); 500 µm (**b**,**c**); 200 µm (d); 10 µm (**e**–**i**).

***Diaporthe gammata*** X.E. Xiao, Crous and H.Y. Li, Persoonia 51: 243 (2023) (Figure 5)

*Description*: Sexual morph not observed. Asexual morph: conidiomata pycnidial, immersed in the bark, erumpent through bark surface, with a single locule. Ectostromatic disc brown to black, with one ostiole per disc, ovoid to circular. Locule undivided, (280–)380–600(–650) μm (av. = 480 μm, n = 20) diam. Conidiophores cylindrical to subcylindrical, hyaline, smooth, aseptate, 5.6–12 × 1–2.6 μm (av. = 8.7 ± 1.8 × 1.8 ± 0.5 μm, n = 50). Conidiogenous cells hyaline, smooth, phialidic, cylindrical, straight or slightly curved, tapered towards the apex, (5–)6–13(–16) × 1–3.5 μm (av. = 8 ± 1.5 × 2 ± 0.5 μm, n = 50). Alpha conidia hyaline, aseptate, fusoid to ovoid, hyaline, aseptate, tapering towards both ends, usually one guttulate at each end, rarely three guttulate, 5.5–9.5 × 1.5–3.7 μm (av. = 7.4 ± 1.2 × 2.5 ± 0.4 μm, n = 50). Beta conidia hyaline, aseptate, filiform, straight or curved, tapering towards both ends, 18–28 × 1.2–1.8 μm (av. = 23 ± 4 × 1.5 ± 0.2 μm, n = 50). Gamma conidia hyaline, aseptate, fusoid to obclavate, multi-guttulate, apex rounded, base rounded to slightly acutate, 6.8–10.6 × 1.8–3 μm (av. = 9 ± 1.3 × 2.4 ± 0.4 μm, n = 50).

*Cultural characteristics*: Colonies initially white, felty with a thicker texture, aerial mycelium lacking, growing up to 36 mm after 3 days, turning grey olivaceous at the centre, becoming dark brick on the surface and sepia to black in the reverse after 12 days (Figure 4b). Conidiomata are distributed randomly at the marginal area.

*Materials examined*: China, Sichuan Province, Guangyuan City: Chaotian District, Walnut Culture Square, 32°40′58″ N, 106°02′08″ E, from branches of *Juglans regia*, 10 October 2023, L. Lin and X.L. Fan (BJFC-S2348, living cultures CFCC 70722 and CFCC 70724); Guangyuan City: Chaotian District, Walnut Culture Square, 32°40′41″ N, 106°02′16″ E, from branches of *Juglans regia*, 10 October 2023, L. Lin and X.L. Fan (BJFC–S2349, living cultures CFCC 70723, 70725); Guangyuan City: Chaotian District, Walnut Culture Square, 32°40′49″ N, 106°02′23″ E, from branches of *Juglans regia*, 10 October 2023, L Lin and X.L. Fan (BJFC-S2350, living culture CFCC 70726).

Notes: *Diaporthe gammata* was first introduced by Xiao et al. (2023) [53] and was isolated from *Citrus reticulata* in Chongqing Municipality, China. It was named for the presence of gamma conidia. Phylogenetically, five isolates form a fully supported clade that are close to *D. gammata* in combined datasets and five gene loci trees (Figure 2, Figures S1–S5). Morphologically, three types of conidia were observed in the present study, which is consistent with the description of *D. gammata* [53]. Therefore, five isolates collected in this study are identified as *D. gammata*. Additionally, this is the first report of *D. gammata* being responsible for *Juglans regia* shoot canker.

**Figure 6:**
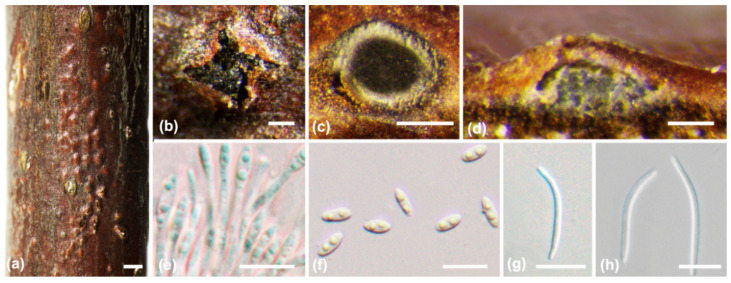
*Diaporthe olivacea* (BJFC-S2351). (**a**–**c**) Habit of conidiomata on twig; (**d**) longitudinal section through a conidioma; (**e**) conidiophores and conidiogenous cell; (**f**) alpha conidia; (**g**,**h**) beta conidia. Scale bars: 2 mm (**a**); 500 µm (**b**,**c**); 200 µm (**d**); 10 µm (**e**–**h**).

***Diaporthe olivacea*** A.L. Jia and X.L. Fan, sp. nov. (Figure 6)

*MycoBank*: MB 854192

*Etymology*: Named after the colour of cultural characteristics, olivaceous buff.

*Typification*: China, Sichuan Province, Guangyuan City: Lizhou District, National Highway 212, 32°26′20″ N, 105°38′26″ E, from branches of Juglans regia, 11 October 2023, L. Lin and X.L. Fan (holotype BJFC-S2351, ex-holotype living culture CFCC 70713). 32°26′25″ N, 105°38′32″ E (paratype BJFC-S2352, ex-paratype living culture CFCC 70715).

*Description*: Sexual morph not observed. Asexual morph: conidiomata pycnidial, immersed in the bark, erumpent through bark surface, with a single locule. Ectostromatic disc black to white, with one ostiole per disc, ovoid to circular. Locule undivided, 400–970 μm (av. = 700 μm, n = 20) diam. Conidiophores hyaline, smooth, 1–2(–3) septate, densely aggregated, cylindrical, 10.8–17.5 × 1–2 μm (av. = 13.4 ± 2 × 1.5 ± 0.3 μm, n = 50). Conidiogenous cells hyaline, smooth, phialidic, cylindrical, tapered towards the apex, 7–18.5 × 1.3–2 μm (av. = 11.5 ± 3 × 1.6 ± 0.2 μm, n = 50). Alpha conidia broadly fusiform to obovoid, hyaline, apex rounded or acute, base acutate, biguttulate to multiguttulate, aseptate, 5.5–9.5 × 1.5–3.7 μm (av. = 7.4 ± 1.2 × 2.5 ± 0.4 μm, n = 50). Beta conidia hyaline, aseptate, filiform, straight or curved, tapering towards both ends, 14–22 × 1.3–2.2 μm (av. = 19 ± 2.5 × 1.7 ± 0.2 μm, n = 50). Gamma conidia are not observed.

*Cultural characteristics*: Colonies covering dish after 7 days in the dark at 25 °C, on the PDA surface with fluffy white aerial mycelium, being hazel on the surface, olivaceous buff in the reverse after 10 days (Figure 4c). Colony margin is regular. Conidiomata are sparse, irregularly distributed over the agar surface after 30 days.

*Additional materials examined*: China, Sichuan Province, Guangyuan City: Lizhou District, National Highway 212, 32°26′28″ N, 105°38′18″ E, from branches of *Juglans regia*, 11 Oct. 2023, L. Lin and X.L. Fan (BJFC-S2353, living cultures CFCC 70714, 70716); 32°26′31″ N, 105°38′22″ E (BJFC-S2354, living culture CFCC 70717).

*Notes*: Phylogenetic analysis combined five gene loci, and each locus showed that all the isolates of *D. olivacea* clustered together in a highly supported clade (ML/BI = 100/1) (Clade 1) and displayed a close relationship, but they were clearly differentiated from *D. musigena*, *D. schimae* and *D. taoicola* (Figure 2, Figures S3–S5). Based on alignments of the separate loci, *D. olivacea* differs from *D. musigena* in ITS, *cal*, *his3*, *tef1-α* and tub2 loci (17/529 for ITS, 35/651 for *cal*, 8/586 for *his3*, 16/691 for *tef1-α* and 25/969 for *tub2*); differs from *D. schimae* in ITS, *cal*, *his3*, *tef1-α* and tub2 loci (10/518 for ITS, 29/678 for *cal*, 2/580 for *his3*, 4/691 for *tef1-α* and 16/969 for *tub2*); and differs from *D. taoicola* in ITS, *tef1-α* and tub2 loci (11/520 for ITS, 8/677 for *tef1-α* and 9/748 for *tub2*). Morphologically, *D. olivacea* differs from *D. musigena* in having smaller conidiophores (10.8–17.5 × 1–2 μm vs. 15–40 × 1.5–2.5 μm) and bigger conidiogenous cells (7–18.5 × 1.3–2 μm vs. 2–5 × 0.5–1 μm) [54]. Moreover, *D. musigena* differs from *D. olivacea* in the production of gamma conidia, which is not observed in *D. olivacea* [55]. *Diaporthe olivacea* differs from *D. schimae* in having smaller alpha conidia (5.5–9.5 × 1.5–3.7 μm vs. 8–8.5 × 2.5–3 μm) and smaller beta conidia (14–22 × 1.3–2.2 μm vs. 27.5–38.5 × 1–1.5 μm) [14], and differs from *D. taoicola* in having smaller alpha conidia (5.5–9.5 × 1.5–3.7 μm vs. 7–9 × 2–3 μm), smaller beta conidia (14–22 × 1.3–2.2 μm vs. 20–25 × 1.5–2 μm) and narrower conidiophores (10.8–17.5 × 1–2 μm vs. 10–25 × 2–3 μm) [11]. 

**Figure 7:**
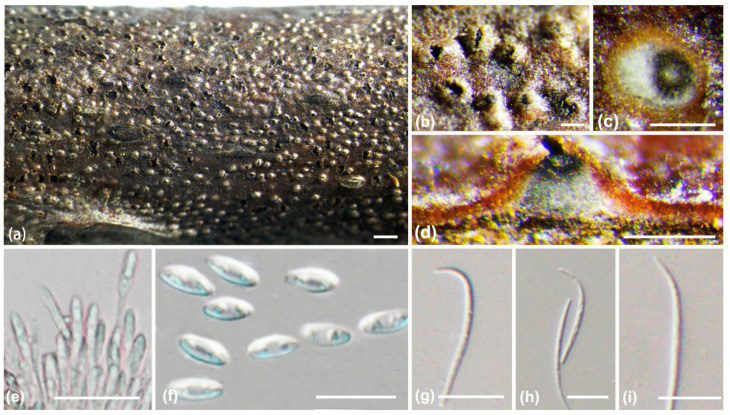
*Diaporthe shangluoensis* (BJFC-S2355); (**a**–**c**) Habit of conidiomata on twig; (**d**) Longitudinal section through a conidioma; (**e**) Conidiophores and conidiogenous cell; (**f**) Alpha conidia; (**g**–**i**) Beta conidia. Scalbars: 2 mm (**a**); 500 µm (**b**,**c**); 200 µm (**d**); 10 µm (**e**–**i**).

***Diaporthe shangluoensis*** A.L. Jia and X.L. Fan, sp. nov. (Figure 7)

*MycoBank*: MB 854193

*Etymology*: Named after the place where the fungus was isolated, Shangluo City.

*Typification*: China, Shaanxi Province, Shangluo City: Shangzhou District, Lijia Plateau Bridge, 33°46′40″ N, 110°06′13″ E, from branches of *Juglans regia*, 13 October 2023, L. Lin and X.L. Fan (holotype BJFC-S2355, ex-holotype living culture CFCC 70728); 33°46′36″ N, 110°06′05″ E (paratype BJFC-S2356, ex-paratype living culture CFCC 70731).

Description: Sexual morph not observed. Asexual morph: conidiomata pycnidial, immersed in the bark, erumpent through bark surface, with a single locule. Ectostromatic disc white to black, with one ostiole per disc, ovoid to circular. Locule undivided, 140–300(–460) μm (av. = 210 μm, n = 20) diam. Conidiophores hyaline, smooth, 1-septate, unbranched, cylindrical, 4.5–9.5 × 1–1.6 μm (av. = 7.5 ± 1.5 × 1.3 ± 0.2 μm, n = 50). Conidiogenous cells hyaline, phialidic, cylindrical, straight or slightly curved, tapered towards the apex, 5–11.5 × 1.5–2.8 μm (av. = 7.5 ± 2 × 1.8 ± 0.3 μm, n = 50). Alpha conidia hyaline, aseptate, fusoid to ovoid, tapering towards both ends, one guttulate at each end, 5.8–8.3 × 2–3 μm (av. = 7 ± 0.5 × 2.7 ± 0.3 μm, n = 50). Beta conidia hyaline, aseptate, filiform, straight or curved, sinuous at one end, tapering towards both ends, eguttulate, 29.5–38 × 1–2 μm, av. = 32.3 ± 2.6 × 1.4 ± 0.1 μm, (n = 50). Gamma conidia are not observed.

*Culture characteristics*: Cultures on PDA incubated at 25 °C in darkness, colony originally flat after 3 days, becoming hazel after 7−10 days (Figure 4d). Colonies flat with a uniform texture, lacking aerial mycelium, margin regular. Conidiomata sparse, irregularly distributed over agar surface after 30 days.

*Additional materials examined*: CHINA, Shaanxi Province, Shangluo City: Shangzhou District, Lijia Plateau Bridge, 33°46′42″ N, 110°06′06″ E, from branches of *Juglans regia*, 13 October 2023, L. Lin and X.L. Fan (BJFC-S2357, living cultures CFCC 70729 and 70730); 33°46′36″ N, 110°06′25″ E (BJFC-S2358, living cultures CFCC 70727 and 70732).

*Notes*: *Diaporthe shangluoensis* was isolated from *Juglans regia* collected in Shaanxi, China. Six isolates are phylogenetically separated from all other available isolates included in this study. *Diaporthe shangluoensis* is most closely related to *D. guangxiensis*, *D*. *hainanensis*, *D. pandanicola*, *D. viciae* and *D. viniferae* (Figure 2, Figures S1–S5), but differentiated from them in ITS (13 different unique fixed alleles by *D. guangxiensis*, 11 by *D*. *hainanensis*, 28 by *D. pandanicola*, 8 by *D. viciae* and 28 by *D. viniferae*), *cal* loci (6 different unique fixed alleles by *D. guangxiensis* and 12 by *D. viniferae*), *his3* loci (4 different unique fixed alleles by *D. viciae*), *tef1-α* loci (20 different unique fixed alleles by *D. guangxiensis*, 21 by *D. hannanensis*, 20 by *D. viciae* and 21 by *D. viniferae*) and *tub2* loci (25 different unique fixed alleles by *D. guangxiensis*, 10 by *D*. *hainanensis*, 8 by *D. pandanicola*, 12 by *D. viciae* and 27 by *D. viniferae*). Moreover, *D. shangluoensis* differs from *D. guangxiensis*, *D. hainanensis* and *D. viniferae* in having bigger beta conidia (29.5–38 × 1–2 vs. 20–32 × 1–1.5 μm) [55], (29.5–38 × 1–2 vs. 23–25 × 1.1 μm) [56], (29.5–38 × 1–2 vs. 23–35 × 1–1.5 μm) [55]. Alpha conidia are smaller than in *D. viciae* (5.8–8.3 × 2–3 vs. 7–10 × 2–4 μm) [57]. Conidiogenous cells are smaller than in *D. hainanensis* (5–11.5 × 1.5–2.8 vs. 14.5–20 × 1.4–1.8 μm) [56]. 

**Figure 8:**
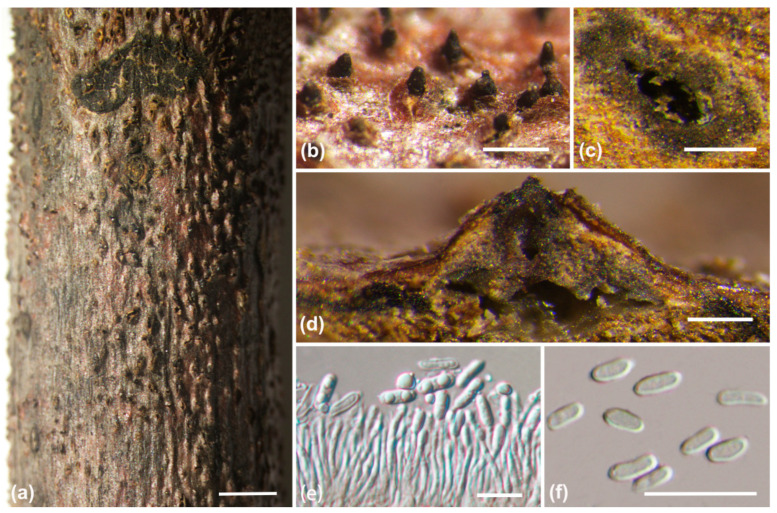
*Diaporthe shangrilaensis* (BJFC-S2359). (**a**–**c**) Habit of conidiomata on twig; (**d**) longitudinal section through a conidioma; (**e**) conidiophores and conidiogenous cell; (**f**) alpha conidia. Scale bars: 2 mm (**a**); 500 µm (**b**,**c**); 200 µm (**d**); 10 µm (**e**,**f**).

***Diaporthe shangrilaensis*** A.L. Jia and X.L. Fan, sp. nov. (Figure 8)

*MycoBank*: MB 854194

*Etymology*: Named after the place where the fungus was isolated, Shangri-La City.

*Typification*: China, Yunnan Province, Diqing Tibetan Autonomous Prefecture, Shangri-La City: Sanba Naxi Township, 27°34′18″ N, 100°1′19″ E, from branches of *Juglans regia*, 9 August 2022, L. Lin (holotype BJFC-S2359, ex-holotype living culture CFCC 70703); 27°34′26″ N, 100°1′5″ E (paratype BJFC-S2360, ex-paratype living culture CFCC 70706).

*Description*: Sexual morph not observed. Asexual morph: conidiomata pycnidial, immersed in the bark, erumpent through bark surface, with a single locule. Ectostromatic disc brown to black, with one ostiole per disc, ovoid to circular. Locule undivided, (70–)110–390(–460) μm (av. = 230 μm, n = 20) diam. Conidiophores hyaline, smooth, aseptate, densely aggregated, cylindrical, straight, 15–20 × 1–2.3 μm (av. = 16 ± 1 × 1.5 ± 0.5 μm, n = 50). Conidiogenous cells hyaline, smooth, branched, phialidic, cylindrical, straight or slightly curved, 4.3–8.6 × 1–2.8 μm (av. = 6 ± 1.2 × 1.8 ± 0.6 μm, n = 50). Alpha conidia hyaline, aseptate, ellipsoidal to cylindrical, obtusely rounded at both ends, usually eguttulate, rarely one guttule at each end, 6–8.8 × 2–3.8 μm (av. = 7.6 ± 0.6 × 2.8 ± 0.4 μm, n = 50). Beta conidia and gamma conidia are not observed.

*Culture characteristics*: Cultures on PDA incubated at 25 °C in darkness, colony originally flat with a white felty aerial mycelium after 3 days, becoming a white compact aerial mycelium at the centre with a smoke-grey aerial mycelium at the margin after 7–10 days (Figure 4e), margin irregular. Conidiomata are sparse, irregularly distributed over the agar surface after 30 days.

*Additional materials examined*: China, Yunnan Province, Diqing Tibetan Autonomous Prefecture, Shangri-La City: Sanba Naxi Township, 27°34′33″ N, 100°1′21″ E, from branches of *Juglans regia*, 9 August 2022, L. Lin (BJFC-S2361, living cultures CFCC 70704 and 70705); 27°34′27″ N, 100°1′16″ E (BJFC-S2362, living cultures CFCC 70707 and 70708).

*Notes*: Based on the combining five gene loci and each locus individually, six isolates representing *D. shangrilaensis* form an independent clade and are phylogenetically distinct from *D. shaanxiensis* in a well-supported clade (ML/BI= 100/1) (Figure 2, Figures S1–S5). *Diaporthe shangrilaensis* can be distinguished from *D. shaanxiensis* based on the differences in ITS, *cal*, *his3* and *tef1-α* loci (18/532 for ITS, 33/702 for *cal*, 25/565 for *his3* and 15/778 for *tef1-α*). In addition, *D. shangrilaensis* differs from *D. shaanxiensis* in having smaller locules (110–390 μm vs. 526–765 μm) and smaller conidiogenous cells (4.3–8.6 × 1–2.8 μm vs. 14.5–17 × 1–1.5 μm) [13].

**Figure 9:**
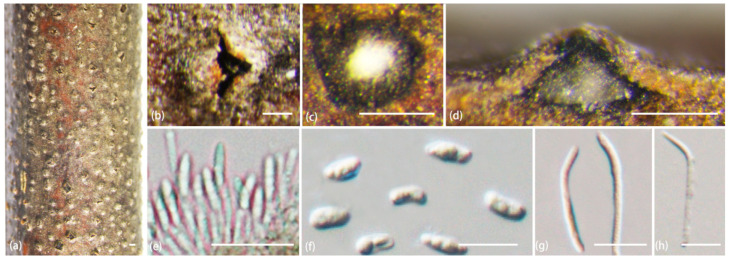
*Diaporthe tibetensis* (BJFC-S2363). (**a**–**c**) Habit of conidiomata on twig; (**d**) longitudinal section through a conidioma; (**e**) conidiophores and conidiogenous cell; (**f**) alpha conidia; (**g**,**h**) beta conidia. Scale bars: 2 mm (**a**); 500 µm (**b**,**c**); 200 µm (**d**); 10 µm (**e**–**h**).

***Diaporthe tibetensis*** C.M. Tian, Qin Yang & X.L. Fan, Mycological Progress 17(7): 847 (2018) (Figure 9)

*Description*: Conidiomata pycnidial, scattered or serried, immersed in bark, discoid to conical, erumpent slightly through the bark surface at maturity, with single locule. Ectostromatic disc brown to black, ovoid to circular. Locule undivided, (370–)420–700(−780) μm (av. = 580 μm, n = 20) diam. Conidiophores hyaline, smooth, aseptate, densely aggregated, cylindrical, 5–20 × 1.5–4 μm. Conidiogenous cells hyaline, smooth, branched, phialidic, cylindrical, tapering towards apex, straight or slightly curved, (1.5–)2–5(–7) × (1–)0.5–1.3(–2) μm (av. = 3.5 ± 0.8 × 1 ± 0.2 μm, n = 50). Alpha conidia abundant in twigs, hyaline, aseptate, ellipsoidal or oval, 0–3–guttulate, 5.8–8 × 2–5 μm (av. = 6.8 ± 0.6 × 2.6 ± 0.5 μm, n = 50). Beta conidia hyaline, aseptate, filiform, straight or curved, tapering towards one end, (8–)10–20(–25) × (0.5–)0.7–1.2(–1.6) μm (av. = 15.5 ± 4.6 × 1 ± 0.3 μm, n = 50). Gamma conidia are not observed.

*Cultural characteristics*: Cultures on PDA incubated at 25 °C in darkness, initially white, irregular. Colony originally flat with a white felty aerial mycelium after 7 days, becoming olivaceous to isabelline with a smoke-grey aerial mycelium after 14 days (Figure 4f), margin irregular, conidiomata sparse, irregularly distributed over agar surface after 30 days.

*Materials examined*: China, Yunnan Province, Diqing Tibetan Autonomous Prefecture, Shangri-La City: Sanba Naxi Township, 27°34′14″ N, 100°1′35″ E, from branches of *Juglans regia*, 9 August 2022, L. Lin (BJFC-S2363 living cultures CFCC 70710 and 70711); 27°34′16″ N, 100°1′33″ E (BJFC-S2364 living cultures CFCC 70709 and 70712); 27°34′25″ N, 100°1′36″ E (BJFC-S2365 living culture CFCC 70702).

*Notes*: *Diaporthe tibetensis* was introduced by Fan et al. (2018) [31] as causing canker disease on *Juglans regia* in Tibet Autonomous Region, China. This species can be distinguished from *D. citrichinensis* and *D. oraccinii* by its shorter conidiogenous cells and larger alpha conidia [31]. In this study, five isolates are grouped together with *D. tibetensis* in combined datasets and five gene loci trees (Figure 2, Figures S1–S5) (ML/BI = 100/1) (Clade 3). Therefore, they are identified as *Diaporthe tibetensis*. Additionally, the current study observed beta conidia of this species.

### 3.3. Analysis of Pathogenicity Test

**Figure 10:**
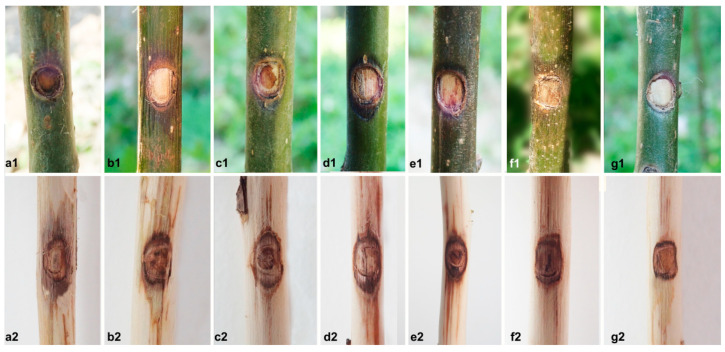
Lesions resulting from inoculation of *Diaporthe* species onto *Juglans regia*, and wound response on the negative control. Disease symptoms inoculated with (**a1**,**a2**) *D. chaotianensis* (CFCC 70720); (**b1**,**b2**) *D. gammata* (CFCC 70722); (**c1**,**c2**) *D. olivacea* (CFCC 70713); (**d1**,**d2**) *D. shangluoensis* (CFCC 70728); (**e1**,**e2**) *D. shangrilaensis* (CFCC 70703); (**f1**,**f2**) *D. tibetensis* (CFCC 70702). (**g1**,**g2**) Blank control. (1) after 1 week, lesions on the bark; (2) after 2 weeks, lesions beneath the bark.

For pathogenicity tests via *Juglans regia* shoot inoculations, the results showed that all the tested *Diaporthe* isolates could induce discoloured and necrotic lesions 14 d post inoculation (Figure 10; Table 2). Sunken cankers were obvious on the stems and produced brown lesions upward and downward from the point of inoculation. At the same time, no lesions were observed on the branches of the control (Figure 10(g1,g2)). Koch’s postulates were fulfilled and confirmed that all the tested *Diaporthe* species in this study are pathogens of *Juglans regia*. *Diaporthe Shangrilaensis* was shown to be the most aggressive species: isolates CFCC 70703, 70705, 70706 of *D. shangrilaensis* caused larger lesions (Figure 10(e1,e2)), followed by the isolates CFCC 70713, 70715, 70716 of *D. olivacea* (Figure 10(c1,c2)), and the remaining isolates CFCC 70728, 70729, 70731 of *D. shangluoensis* induced smaller lesions (Figure 10(d1,d2)). Isolates of *D. gammata* caused only slight discolouration around the inoculation points (Figure 10(b1,b2)). In contrast, the remaining *Diaporthe* isolates induced very limited lesions (5 < mean lesion length < 6 mm). Although the difference in mean lesion length between *D. chaotianensis* and *D. tibetensis* was not significant, the disease incidence of *D. chaotianensis* (89%) was higher than that of *D. tibetensis* (56%). Therefore, *D. chaotianensis* was found to be more virulent than *D. tibetensis*. *Diaporthe gammata* was significantly lower than the other five species in lesion length, with canker length averaging 9.1 ± 0.6 mm. ANOVA revealed significant (*p* < 0.05) differences among the treatment means in all six species (Table 2).

## 4. Discussion

The current study reveals the diversity and pathogenicity of *Diaporthe* species from *Juglans regia* in China. Six *Diaporthe* species were identified from the collected specimens in this study (*Diaporthe chaotianensis*, *D. gammata*, *D. olivacea*, *D. shangluoensis D. shangrilaensis* and *D. tibetensis*). Among these, *D. chaotianensis*, *D. olivacea*, *D. shangluoensis* and *D. shangrilaensis* were described as four novel species and *D. gammata* was reported for the first time in *Juglans regia* trees. These findings suggest a higher level of diversity among *Diaporthe* species responsible for cankers on *Juglans regia* than has been previously recognized. Moreover, Koch’s postulates confirmed that those species were pathogens of *Juglans regia*.

The taxonomy of *Diaporthe* species is increasingly attracting attention from researchers. Most research focuses on the identification and descriptions of novel species and new host records, as well as on the regulation of pathogenicity in important *Diaporthe* species, indicating that genus *Diaporthe* has a high potential for rapid evolution [14]. Previous studies have revealed that *Diaporthe* species have high genetic diversity on a single host. For example, Gao et al. (2016) [58] reported nine species of *Diaporthe* isolated from *Camellia* in China, and Wan et al. (2023) [59] revealed three new *Diaporthe* species on *Acer palmatum* in China [58,59]. This study collected an extensive number of *Diaporthe* isolates from areas of *Juglans regia* cultivation to reveal the genetic diversity of *Diaporthe* species. Previous studies have reported the presence of *Diaporthe* species in *Juglans regia*, and the results of this study further support these findings. Indeed, more novel species will likely be found in the future because several species of *Diaporthe* have a wide host range and can move between hosts among geographic regions. For example, *D. gammata* was originally reported on *Citrus* in Chongqing Municipality [53], but the fungus has also been found on *Juglans regia* in this study.

The current pathogenicity tests showed that *D. chaotianensis*, *D. gammata*, *D. olivacea*, *D. shangluoensis*, *D. shangrilaensis* and *D. tibetensis* are pathogens, which could pose threats to the *Juglans regia* industry in China. Furthermore, *D. shangrilaensis*, *D. olivacea* and *D. shangluoensis* are more aggressive among the six species, and this should be considered in the development of disease control measures. Among these species, *Diaporthe shangrilaensis* demonstrated greater aggressiveness, evidenced by its more potent virulence and the larger lesions it induced. To clarify the reproductive characteristics of this pathogen, additional quantitative experimental analysis is warranted. The virulence of *Diaporthe* species could be affected by environmental factors such as moisture content, rainfall intensity and temperature [60,61]. Manawasinghe et al. (2018) [15] found that environmental factors could alter the life mode of the fungi, from endophytic or saprophytic to pathogenic, thus enabling the colonization of new hosts. *Diaporthe tibetensis* was first reported by Fan et al. (2018) [31] on *Juglans regia* in Tibet Autonomous Region, and was also found on *Juglans regia* branches in Yunnan in this study, where the climates are quite different from the place where the pathogenicity tests were conducted. The pathogenicity tests showed that the majority of these species obtained in this study are weakly aggressive or non-aggressive to *Juglans regia* branches (Figure 10(f1,f2); Table 2). This may be because the experimental conditions were different from those in the natural environment and because of differences in climate between the north and south of China. This implies that *D. tibetensis* may become highly aggressively pathogenic to *Juglans regia* under favourable environmental conditions, which need to be determined by further research in order to prevent *Juglans regia* cankers.

This study provides novel information on the ability of those species to cause disease in *Juglans regia*. In addition, many studies have reported that most *Diaporthe* species had a wide host range. For example, *Diaporthe eres* could cause shoot blight, leaf necrosis and branch canker on different hosts [62,63,64,65,66]. *Diaporthe sojae* was confirmed as the pathogen of fungal diseases on pears, sunflowers, honeybush, kiwi fruit and soybeans [16,67,68,69,70]. Those studies indicated that species obtained in this study may be capable of infecting other plants. In this study, pathogenicity tests were conducted exclusively on *Juglans regia*. Therefore, it would be recommended that pathogenicity tests be conducted on other plants in future studies. In addition, among the eight species from *Juglans regia* in China previously reported, only *D. amygdali* was confirmed as a pathogen causing twig canker disease in *Juglans regia* [30]. The present study confirms that *D. tibetensis* is also a causal agent on *Juglans regia*. The pathogenicity of most reported species has not been tested, and their potential economic impact on *Juglans regia* remains unknown. Thus, the pathogenicity of *Diaporthe* species from *Juglans regia* needs to be studied further. 

In conclusion, this study has focused on the diversity and pathogenicity of *Diaporthe* species from *Juglans regia* in China. Six species were isolated, and pathogenicity tests indicated that they exhibited varying degrees of pathogenicity. The current findings establish a foundational understanding crucial for enhancing disease management strategies. As for canker disease in *Juglans regia* trees, future studies should focus on the most widespread and aggressive pathogens reported here, and prevention and control measures should be investigated to mitigate the impact of canker diseases.

## Figures and Tables

**Table 2:** Disease incidence and lesion size on *Juglans regia* branches one month after inoculation with isolates of *Diaporthe* species.

Species	Isolate	Disease Incidence (%)	Lesion Size (mm)
*Diaporthe chaotianensis*	CFCC 70718–70720	89	13.6 ± 0.8 d
*Diaporthe gammata*	CFCC 70722–70724	39	9.1 ± 0.6 e
*Diaporthe olivacea*	CFCC 70713, 70715, 70716	56	21.4 ± 1.1 b
*Diaporthe shangluoensis*	CFCC 70728, 70729, 70731	61	17.6 ± 1.7 c
*Diaporthe shangrilaensis*	CFCC 70703, 70705, 70706	94	31.3 ± 1.5 a
*Diaporthe tibetensis*	CFCC 70702, 70710, 70711	56	13.3 ± 0.5 d
Control	Noncolonized potato dextrose agar plug	0	6.5 ± 0.2 f

Different lowercase letters indicate significant differences among six fungal species (LSD test, *p* < 0.05).

## Data Availability

All sequence data are available in NCBI GenBank following the accession numbers in the manuscript.

## Supplementary Materials

The following supporting information can be downloaded at: https://www.mdpi.com/article/10.3390/jof10080583/s1, Table S1: Strains and their GenBank accession numbers used in the molecular phylogenetic analyses of *Diaporthe*. Figure S1: Phylogenetic tree of *Diaporthe* based on ITS loci. Figure S2: Phylogenetic tree of *Diaporthe* based on *cal* loci. Figure S3: Phylogenetic tree of *Diaporthe* based on *his3* loci. Figure S4: Phylogenetic tree of *Diaporthe* based on *tef1-α* loci. Figure S5: Phylogenetic tree of *Diaporthe* based on *tub2* loci.
