# Supplementary material for: Diversity and Pathogenicity of Six Diaporthe Species from Juglans regia in China
Source: J Fungi (Basel). 2024 Aug 16;10(8):583. doi: 10.3390/jof10080583 (PMC11355219; doi:10.3390/jof10080583)
Supplement: Supplementary file 1 [file jof-10-00583-s001.zip › Supplementary Files/Table S1.pdf]

**Table S1.** Strains and their GenBank accession numbers used in the molecular phylogenetic analyses of *Diaporthe*. Newly generated sequences are in bold. (T) = ex–type strain.

| Species | Strain | Host | Origin | GenBank accession numbers | | | | |
|---|---|---|---|---|---|---|---|---|
| | | | | ITS | *cal* | *his3* | *tef1-α* | *tub2* |
| *Diaporthe acaciarum* | CBS138862T | *Acacia tortilis* | Tanzania | KP004460 | – | KP004504 | – | KP004509 |
| *D. acaciigena* | CBS 129521T | *Acacia retinodes* | Australia | KC343005 | KC343247 | KC343489 | KC343731 | KC343973 |
| *D. acericola* | MFLUCC17-0956T | *Acer negundo* | Italy | KY964224 | KY964137 | – | KY964180 | KY964074 |
| *D. acerigena* | CFCC 52554T | *Acer tataricum* | China | MH121489 | MH121413 | MH121449 | MH121531 | – |
| *D. acerina* | CBS 137.27 | *Acer negundo* | – | KC343006 | KC343248 | KC343490 | KC343732 | KC343974 |
| *D. acuta* | PSCG 047T | *Pyrus pyrifolia* | China | MK626957 | MK691125 | MK726161 | MK654802 | MK691225 |
| *D. acutispora* | CGMCC 3.18285T | *Coffea* sp. | China | KX986764 | KX999274 | KX999235 | KX999155 | KX999195 |
| *D. aestuarium* | BRIP 59930aT | *Callistachys lanceolata* | Australia | 0M918686 | – | – | OM960595 | 0M960613 |
| *D. africane* | CBS150080T | *Polyscias fulva* | Tonga | OR198681 | OR225233 | OR225231 | OR225227 | OR225229 |
| *D. afzeliae* | SDBR-CMU467T | *Afzelia xylocarpa* | Thailand | 0Q600199 | 0Q646882 | 0Q646886 | 0Q603502 | 0Q678279 |
| *D. aitkeniae* | BRIP 58827aT | Unknown | Australia | OR019750 | – | – | OR039640 | OR039647 |
| *D. alangii* | CFCC 52556T | *Alangium kurzii* | China | MH121491 | MH121415 | MH121451 | MH121533 | MH121573 |
| *D. albosinensis* | CFCC 53066T | *Betula albosinensis* | China | MK432659 | MK442979 | MK443004 | MK578133 | MK578059 |
| *D. alleghaniensis* | CBS 495.72T | *Betula alleghaniensis* | Canada | FJ889444 | KC343249 | KC343491 | GQ250298 | KC843228 |
| *D. alnea* | CBS 146.46T | *Alnus* sp. | Netherlands | KC343008 | KC343250 | KC343492 | KC343734 | KC343976 |
| *D. ambigua* | CBS114015T | *Pyrus communis* | South Africa | KC343010 | KC343252 | KC343494 | KC343736 | KC343978 |
| *D. ampelina* | CBS 114016T | *Vitis vinifera* | France | AF230751 | JX197443 | – | GQ250351 | JX275452 |
| *D. amygdali* | CBS 126679T | *Prunus dulcis* | Portugal | KC343022 | KC343264 | KC343506 | KC343748 | KC343990 |
| *D. amygdali* | CGMCC 3.15183 | *Ternstroemia* | China | KC153098 | – | – | KC153089 | – |
| *D. anacardii* | CBS 720.97T | *Anacardium occidentale* | East Africa | KC343024 | KC343266 | KC343508 | KC343750 | KC343992 |
| *D. angelicae* | CBS111592T | *Heracleum sphondylium* | Austria | KC343026 | KC343268 | KC343511 | KC343752 | KC343994 |
| *D. angustiapiculata* | ZHKUCC 22-0184 | *Phoenix roebelenii* | China | OR164920 | OR166299 | – | OR166269 | OR166309 |
| *D. angustiapiculata* | ZHKUCC 22-0194 | *Phoenix roebelenii* | China | OR164921 | OR166300 | – | OR166270 | OR166310 |
| *D. anhuiensis* | CNUCC 201902T | *Cunninghamia lanceolata* | China | MN219727 | MN224556 | MN224550 | MN224669 | MN227009 |
| *D. annellsiae* | BRIP 59731aT | *Mangifera indica* | Australia | 0M918687 | – | – | 0M960596 | 0M960614 |
| *D. antonovae* | BRIP 58824bT | *Helianthus annuus* | Australia | OR019751 | – | – | OR039641 | OR039648 |
| *D. apiculata* | LC 3418T | *Camellia sinensis* | China | KP267896 | – | KP293550 | KP267970 | KP293476 |
| *D. aquatica* | IFRDCC 3051T | *Aquatic habitat* | China | JQ797437 | – | – | – | – |
| *D. araliae-chinensis* | GUCC 412.17 | *Aralia chinensis* | China | OP581220 | – | – | OP688525 | OP688550 |
| *D. araucanorum* | CBS145285T | *Araucaria araucana* | Chile | MN509711 | MN974277 | – | MN509733 | MN509722 |
| *D. arctii* | CBS 136.25 | *Arctium sp.* | Unknown | KC343031 | KC343273 | KC343515 | KC343757 | KC343999 |
| *D arecae* | CBS 161.64T | *Areca catechu* | India | KC343032 | KC343274 | KC343516 | KC343758 | KC344000 |
| *D. arengae* | CBS 114979T | *Arenga engleri* | Hong Kong | KC343034 | KC343276 | KC343518 | KC343760 | KC344002 |
| *D. arezzoensis* | MFLU 19-2880T | *Cytisus* sp. | Italy | MT185503 | – | – | – | MT454055 |
| *D. aseana* | MFLUCC 12-0299aT | Unknown | Thailand | KT459414 | KT459464 | – | KT459448 | KT459432 |
| *D. asheicola* | CBS 136967T | *Vaccinium ashei* | Chile | KJ160562 | KJ160542 | – | KJ160594 | KJ160518 |
| *D. aspalathi* | CBS117169T | *Aspalathus linearis* | South Africa | KC343036 | KC343278 | KC343520 | KC343762 | KC344004 |
| *D. atlantica* | CECT 21217T | *Festuca rubra subsp. pruinosa* | Spain | 0N159893 | ON364019 | ON398810 | ON398831 | ON364040 |
| *D. australafricana* | CBS111886T | *Vitis vinifera* | Australia | KC343038 | KC343280 | KC343522 | KC343764 | KC344006 |
| *D. australiana* | BRIP 66145T | *Macadamia* sp. | Australia | MN708222 | – | – | MN696522 | MN696530 |
| *D. australpacifica* | BRIP 60163dT | *Amaranthus blitum* | Australia | 0M918688 | – | – | OM960597 | 0M960615 |
| *D. averrhoae* | SCHM 3605T | *Averrhoae carambolae* L. | China | AY618930 | – | – | – | – |
| *D. baccae* | CBS 136972 | *Vaccinium corymbosum* | Italy | KJ160565 | – | MF418264 | KJ160597 | MF418509 |
| *D. batatas* | CBS 122.21 | *Ipomoea batatas* | USA | KC343040 | KC343282 | KC343524 | KC343766 | KC344008 |
| *D. bauhiniae* | CFCC 53071T | *Bauhinia purpurea* | China | MK432648 | MK442970 | MK442995 | MK578124 | MK578051 |
| *D. beasley* | BRIP 59326aT | *Psidium guajava* | Australia | 0M918689 | – | | OM960598 | 0M960616 |
| *D. beckhausii* | CBS 138.27 | *Viburnum sp.* | Unknown | KC343041 | KC343283 | KC343525 | KC343767 | KC344009 |
| *D. beilharziae* | BRIP 54792T | *Indigofera australis* | Australia | JX862529 | – | – | JX862535 | KF170921 |
| *D. benedicti* | ATCC MYA-4970T | *Salix* sp. | California | KM669929 | KM669862 | – | KM669785, | – |
| *D. berteroae* | BRIP 57900aT | *Helianthus annuus* | Australia | OR019752 | – | – | OR039642 | OR039649 |
| *D. hetulae* | CFCC 50469T | *Betula platyphylla* | China | KT732950 | KT732997 | KT732999 | KT733016 | KT733020 |
| *D. betulicola* | CFCC 51128T | *Betula albosinensis* | China | KX024653 | KX024659 | KX024661 | KX024655 | KX024657 |
| *D. betulina* | CFCC 525621 | *Betula albosinensis* | China | MH121497 | MH121421 | MH121457 | MH121539 | MH121579 |

| | | | | | | | | |
|---|---|---|---|---|---|---|---|---|
| *D. biconispora* | CGMCC 3.17252T | *Citrus grandis* | China | KJ490597 | – | KJ490539 | KJ490476 | KJ490418 |
| *D. biguttulata* | ZJUD47 | *Citrus limon* | China | KJ490582 | | KJ490524 | KJ490461 | KJ490403 |
| *D. biguttulata* | ZJUD48 | *Citrus limon* | China | KJ490583 | | KJ490525 | KJ490462 | KJ490404 |
| *D bohemiae* | CBS143347T | *Vitis yinifera* | Czech Republic | MG281015 | MG281710 | MG281361 | MG281536 | MG281188 |
| *D. bombacis* | SDBR-CMU468 | *Bombax ceiba* L. | Thailand | 0Q600198 | 0Q646881 | 0Q646885 | 0Q603501 | 0Q678278 |
| *D. bounty* | BRIP 59361a' | *Malus domestica* | Australia | 0M918690 | – | – | OM960599 | 0M960617 |
| *D. brasiliensis* | CBS 133183T | *Aspidosperma tomentosum* | Brazil | KC343042 | KC343284 | KC343526 | KC343768 | KC344010 |
| *D. breyniae* | CBS 148910T | *Breynia oblongifolia* | Cameroon | 0N400846 | ON409189 | ON409187 | ON409188 | 0N409186 |
| *D. brideliae* | CBS 148911T | *Bridelia ndellensis* | Cameroon | OR348649 | OR468837 | OR468807 | OR468817 | OR468827 |
| *D. browniae* | BRIP 27613a | *Sorghum leiocladium* | Australia | OR122282 | – | – | OR135525 | OR135526 |
| *D. brumptoniae* | BRIP 59403aT | *Amaranthus* sp. | Australia | 0M918702 | – | – | 0M960611 | 0M960629 |
| *D. butterlyi* | BRIP 59194aT | *Rapistrum rugosum* | Australia | OR019753 | – | – | OR039643 | OR039650 |
| *D. caatingaensis* | CBS 141542T | *Tacinga inamoena* | Brazil | KY085927 | KY115597 | KY115605 | KY115603 | KY115600 |
| *D. cameroonensis* | CBS 148913T | *Atractogyne gabonii* | Cameroon | OR348650 | OR468836 | OR468806 | OR468816 | OR468826 |
| | STMA 18289 | *Atractogyne gabonii* | Cameroon | 0R348651 | OR468835 | OR468805 | OR468815 | OR468825 |
| | STMA 18290 | *Atractogyne gabonii* | Cameroon | 0R348652 | OR468834 | OR468804 | OR468814 | OR468824 |
| *D. camelliae-oleiferae* | HNZZ 027T | *Camellia oleifera* | China | MZ509555 | MZ504685 | MZ504696 | MZ504707 | MZ504718 |
| *D. camelliae-sinensis* | SAUCC 194.92T | *Camellia sinensis* | China | MT822620 | MT855699 | MT855588 | MT855932 | MT855817 |
| *D. camporesii* | JZB 320143T | *Urtica dioidca* | taly | MN533805 | – | – | – | MN561316 |
| *D. canthii* | CBS 132533T | *Canthium inerne* | South Africa | JX069864 | KC843174 | – | KC843120 | KC843230 |
| *D. careyae* | SDBR-CMU469T | *Careya sphaerica* | Thailand | 0Q600196 | 0Q646879 | 0Q646883 | – | 0Q678276 |
| *D. carpini* | CBS114437 | *Carpinus betulus* | Sweden | KC343044 | KC343286 | KC343528 | KC343770 | KC344012 |
| *D. carriae* | BRIP 59932aT | *Verticordia grandis* | Australia | OM918691 | – | – | OM960600 | 0M960618 |
| *D. caryae* | CFCC 52563T | *Carya illinoinensis* | China | MH121498 | MH121422 | MH121458 | MH121540 | MH121580 |
| *D. cassines* | CBS 136440T | *Cassine peragua* | South Africa | KF777155 | – | – | KF777244 | – |
| *D. caulivora* | CBS 127268 | *Glycine max* | Croatia | KC343045 | KC343287 | KC343529 | KC343771 | KC344013 |
| *D. celastrina* | CBS 139.27T | *Celastrus* sp. | USA | KC343047 | KC343289 | KC343531 | KC343773 | KC344015 |
| *D. celeris* | CBS 143349T | *Vitis vinifera* | United Kingdom | MG281017 | MG281712 | MG281363 | MG281538 | MG281190 |
| *D. celticola* | CFCC 53074T | *Celtis vandervoetiana* | China | MK573948 | MK574587 | MK574603 | MK574623 | MK574643 |
| *D. celtidis* | NCYU 19-0357T | *Celtis formosana* | China | MW114346 | – | – | MW192209 | MW148266 |
| *D. ceratozamiae* | CBS 131306I | *Ceratozamia robusta* | Australia | JQ044420 | – | – | – | – |
| *D. cercidis* | CFCC 52565T | *Cercis chinensis* | China | MH121500 | MH121424 | MH121460 | MH121542 | MH121582 |
| *D. cerradensis* | CMRP 4331T | *Stryphnodendron adstringens* | Brazil | MN173198 | MW751655 | MW751663 | MT311685 | MW751671 |
| *D. cf.heveae 1* | CBS 852.97 | *Hevea brasiliensis* | Brazil | KC343116 | KC343358 | KC343600 | KC343842 | KC344084 |
| *D. cf.heveae 2* | CBS 681.84 | *Hevea brasiliensis* | Brazil | KC343117 | KC343359 | KC343601 | KC343843 | KC344085 |
| *D. chamaeropis* | CBS 454.81 | *Chamaerops humilis* | Greece | KC343048 | KC343290 | KC343532 | KC343774 | KC344016 |
| *D. changpingensis* | CFCC 58812T | *Robinia pseudoacacia* | China | OQ912925 | OQ910202 | OQ910234 | OQ910264 | OQ910292 |
| *D. changpingensis* | CFCC 58813 | *Robinia pseudoacacia* | China | OQ912926 | OQ910203 | OQ910235 | OQ910265 | OQ910293 |
| ***D. chaotianensis*** | **CFCC 70718** | ***Juglans regia* L.** | **China** | **PP862881** | **PP868509** | **PP868597** | **PP868535** | **PP868566** |
| ***D. chaotianensis*** | **CFCC 70719** | ***Juglans regia* L.** | **China** | **PP862882** | **PP868510** | **PP868598** | **PP868536** | **PP868567** |
| ***D. chaotianensis*** | **CFCC 70720T** | ***Juglans regia* L.** | **China** | **PP862883** | **PP868511** | **PP868599** | **PP868537** | **PP868568** |
| ***D. chaotianensis*** | **CFCC 70721** | ***Juglans regia* L.** | **China** | **PP862884** | **PP868512** | **PP868600** | **PP868538** | **PP868569** |
| *D. charlesworthii* | BRIP 54884mT | *Rapistrum rugostrum* | Australia | KJ197288 | – | – | KJ197250 | KJ197268 |
| *D. chensiensis* | CFCC 52567T | *Abies chensiensis* | China | MH121502 | MH121426 | MH121462 | MH121544 | MH121584 |
| *D. chiangmaiensis* | MFLUCC18-0544T | *Alstonia scholaris* | Thailand | 0K393703 | – | – | 0L439483 | – |
| *D. chimonanthi* | SCHM3614 | *Elaeagnus conferta* | China | AY622993 | – | – | – | – |
| *D chinensis* | MFLUCC 19-0101T | *Magnolia candolli* | China | MW187324 | MW294199 | – | MW205017 | MW245013 |
| *D. chonggingensis* | PSCG 435T | *Pyrus pyrifolia* | China | MK626916 | MK691209 | MK726257 | MK654866 | MK691321 |
| *D. chromolaenae* | MFLUCC17-1422T | *Chromolaena odorata* | Thailand | MH094275 | – | – | – | – |
| *D. chrysalidocarp* | SAUCC 194.35T | *Chrysalidocarpus lutescens* | China | MT822563 | MT855646 | MT855532 | MT855876 | MT855760 |
| *D. cichorii* | MFLUCC17-1023T | *Cichorium intybus* | ltaly | KY964220 | KY964133 | – | KY964176 | KY964104 |
| *D. cinnamom* | CFCC 52569T | *Cinnamomum* sp. | China | MH121504 | – | MH121464 | MH121546 | MH121586 |
| *D. cinerascens* | CBS719.96 | *Ficus carica* | Bulgaria | KC343050 | KC343292 | KC343534 | KC343776 | KC344018 |
| *D. cissampel* | CBS 141331T | *Cissampelos capensis* | South Africa | KX228273 | – | KX228366 | – | KX228384 |
| *D. citri* | CBS 135422T | *Citrus* sp. | USA | KC843311 | KC843157 | MF418281 | KC843071 | KC843187 |
| *D. citriasiana* | CBS 134240T | *Citrus unshiu* | China | JQ954645 | KC357491 | MF418282 | J0954663 | KC357459 |
| *D. citrichinensis* | CBS134242T | *Citrus unshiu* | China | JQ954648 | KC357494 | KJ420880 | JQ954666 | MF418524 |
| *D. clematidina* | MFLUCC 17-2060T | *Clematis subumbellata* | Thailand | MT310657 | MT394624 | – | MT394669 | MT394623 |
| *D. collariana* | MFLUCC 17-2636T | *Magnolia champaca* L. | Thailand | MG806115 | MG783042 | – | MG783040 | MG783041 |
| *D. compacta* | LC3083T | *Camellia sinensis* | China | KP267854 | – | KP293508 | KP267928 | KP293434 |

| | | | | | | | | |
|---|---|---|---|---|---|---|---|---|
| *D. conica* | CFCC 52571T | *Alangium chinense* | China | MH121506 | MH121428 | MH121466 | MH121548 | MH121588 |
| *D. constancestone* | BRIP 52819b | *Acacia* sp. | Australia | OR290124 | – | – | OR335743 | OR352592 |
| *D. constrictospora* | CGMCC 3.20096T | Unknown | China | MT385947 | MT424718 | MW022487 | – | MT424702 |
| *D. convolvuli* | CBS 124654 | *Convolvulus arvensis* | Turkey | KC343054 | KC343296 | KC343538 | KC343780 | KC344022 |
| *D. coryli* | CFCC 53083T | *Corylus mandshurica* | China | MK432661 | MK442981 | MK443006 | MK578135 | MK578061 |
| *D. corylicola* | CFCC 53986T | *Corylus heterophylla* | China | MW839880 | MW836684 | MW836717 | MW815894 | MW883977 |
| *D. crataegi* | CBS114435 | *Crataegus rhipidophylla* | Sweden | KC343055 | KC343297 | KC343539 | KC343781 | KC344023 |
| *D crotalariae* | CBS 162.33T | *Crotalaria spectabilis* | USA | KC343056 | KC343298 | KC343540 | KC343782 | KC344024 |
| *D. crousii* | CAA823T | *Vaccinium corymbosum* | Portugal | MK792311 | MK883835 | MK871450 | MK828081 | MK837932 |
| *D. cucurbitae* | DAOM 42078T | *Cucumis* sp. | Canada | KM453210 | – | KM453212 | KM453211 | KP118848 |
| *D. cuppatea* | CBS 117499T | *Aspalathus linearis* | South Africa | AY339322 | JX197414 | KC343541 | AY339354 | JX275420 |
| *D. cyatheae* | YMJ 1364T | *Cyathea lepifera* | China | JX570889 | KC465410 | – | KC465406 | KC465403 |
| *D. cylindriformispora* | MFLU 18-2292 | Unknown | China | OR225015.1 | – | – | OR140382.1 | OR189510.1 |
| *D. cynaroidis* | CBS122676 | *Protea cynaroides* | South Africa | KC343058 | KC343300 | KC343542 | KC343784 | KC344026 |
| *D. cytosporella* | CBS 137020T | *Citrus limon* | Spain | KC843307 | KC843141 | MF418283 | KC843116 | KC843221 |
| *D. decedens* | CBS109772 | *Corylus avellana* | Austria | KC343059 | KC343301 | KC343543 | KC343785 | KC344027 |
| *D. delonicis* | MFLU 16-1059T | *lpomoea batatas* | China | MT215490 | – | – | – | MT212209 |
| *D. dejiangensis* | GUCC 421.2T | *Juglans regia* L. | China | OP581221 | – | – | OP688526 | OP688551 |
| *D detrusa* | CBS109770 | *Berberis vulgaris* | Austria | KC343061 | KC343303 | KC343545 | KC343787 | KC344029 |
| *D. diospyricola* | CBS 136552T | *Diospyros whyteana* | South Africa | KF777156 | – | – | – | – |
| *D. diospyrina* | CFCC 58820T | *Diospyros kaki* | China | OQ912929 | OQ910206 | OQ910236 | OQ910268 | OQ910296 |
| *D. diospyrina* | CFCC 58821 | *Diospyros kaki* | China | OQ912930 | OQ910207 | OQ910237 | OQ910269 | OQ910297 |
| *D discoidispora* | ICMP 20662T | *Citrus unshiu* | China | KJ490624 | – | KJ490566 | KJ490503 | KJ490445 |
| *D. donglingensis* | CFCC 58806 | *Corylus heterophylla* | China | OQ912931 | – | OQ910238 | OQ910270 | OQ910298 |
| *D. donglingensis* | CFCC 58807 | *Corylus heterophylla* | China | OQ912932 | – | OQ910239 | OQ910271 | OQ910299 |
| *D. drenthii* | BRIP 66524T | *Macadamia* sp. | Australia | MN708229 | – | – | MN696526 | MN696537 |
| *D. durionigena* | VTCC 930005T | *Durio zibethinus* | Vietnam | MN453530 | – | – | MT276157 | MT276159 |
| *D. elaeagni-confertae* | SAUCC194.47T | *Elaeagnus conferta* Roxb. | China | MT822575 | MT855656 | MT855544 | MT855888 | MT855772 |
| *D. elaeagni-glabrae* | CGMCC 3.18287T | *Elaeagnus glabra* | China | KX986779 | KX999281 | KX999251 | KX999171 | KX999212 |
| *D. eleagni* | CBS 504.72 | *Elaeagnus* sp. | Netherlands | KC343064 | KC343306 | KC343548 | KC343790 | KC344032 |
| *D. elizabethandersoniae* | BRIP 59733a | *Hyocereus* | Australia | OR290125 | – | – | OR335744 | OR352593 |
| *D. elizabethblackwelliae* | BRIP 74769a | *Cyperus aromaticus* | Australia | OR290128 | – | – | OR335747 | OR352596 |
| *D. ellipsospora* | CGMCC 3.20099T | Unknown | China | MT385949 | MT424720 | MW022488 | MT424684 | MT424704 |
| *D. endocitricola* | ZHKUCC 20-0012T | *Citrus grandis* | China | MT355682 | MT409312 | – | MT409336 | MT409290 |
| *D. endophytica* | CBS 133811T | *Schinus terebinthifolius* | Brazil | KC343065 | KC343307 | KC343549 | KC343791 | KC344033 |
| *D. eres* | CBS 138594T | *Ulmus* sp. | Germany | KJ210529 | KJ434999 | KJ420850 | KJ210550 | KJ420799 |
| *D. eres* | CFCC 51632(type strain of D.camptothecicola) | *Camptotheca acuminata* Decne. | China | KY203726 | KY228877 | KY228881 | KY228887 | KY228893 |
| *D. eres* | CGMCC 3.17089(type strain of D.longicicola | *Lithocarpus glabra* | China | KF576267 | – | – | KF576242 | KF576291 |
| *D. eres* | MFLUCC16-0113 (type strain of D.momicola) | *Prunus persica* | China | KU557563 | KU557611 | – | KU557631 | KU557587 |
| *D. eres* | CGMCC 3.15181 (strain originally named D. mahothocarpi Nom.Inval. | *Lithocarpus glabra* | China | KC153096 | – | – | KC153087 | – |
| *D. eres* | CGMCC 3.17084(type strain of D.ellipicola) | *Lithocarpus glabra* | China | KF576270 | – | – | KF576245 | KF576291 |
| *D. eres* | CGMCC 3.17081 (type strain of D.biguttusis) | *Lithocarpus glabra* | China | KF576282 | – | – | KF576257 | KF576306 |
| *D. etinsideae* | BRIP 64096aT | *Annona muricata* | Australia | OM918692 | – | – | OM960601 | 0M960619 |
| *D. eucalyptorum* | CBS 132525T | *Eucalyptus* sp. | China | JX069862 | – | – | – | – |
| *D. eucommiigena* | GUCC 420.19 | *Eucommia ulmoides* | China | OP581224 | – | – | OP688529 | OP688554 |
| *D. eucommiigena* | GUCC 420.9 | *Eucommia ulmoides* | China | OP581223 | – | – | OP688528 | OP688553 |
| *D. eugeniae* | CBS 444.82 | *Eugenia aromatica* | Indonesia | KC343098 | KC343340 | KC343582 | KC343824 | KC344066 |
| *D. fibrosa* | CBS 109751 | *Rhamnus cathartica* | Austria | KC343099 | KC343341 | KC343583 | KC343825 | KC344067 |

| | | | | | | | | |
|---|---|---|---|---|---|---|---|---|---|
| *D. fici-septicae* | MFLU 18-2588T | *Ficus septica* | China | MW114348 | – | – | MW192211 | MW148268 |
| *D. foeniculina* | CBS 111553T | *Foeniculum vulgare* | Spain | KC343101 | KC343343 | KC343585 | KC343827 | KC344069 |
| *D. fohaiensis* | SAUCC194.113T | *Lithocarpus fohaiensis* | China | MT822641 | MT855720 | MT855608 | MT855953 | MT855838 |
| *D. fohaiensis* | SAUCC194.115 | *Lithocarpus fohaiensis* | China | MT822643 | MT855722 | MT855610 | MT855955 | MT855840 |
| *D. foikelawen* | CBS 145289T | *Drimys winteri* | Chile | MN509713 | MN974278 | – | MN509735 | MN509724 |
| *D. folicola* | CFCC 55351d | *Acer palmatum* | China | MZ560718 | MZ577305 | MZ577296 | MZ577278 | MZ577287 |
| *D. forlicesenica* | MFLUCC 17-1015T | *Dorycnium hirsutum* | Italy | KY964215 | – | – | KY964171 | KY964099 |
| *D. fraxini-angustifoliae* | BRIP 54781T | *Fraxinus angustifolia* | Australia | JX862528 | – | – | JX852534 | KF170920 |
| *D. fraxinicola* | CFCC 52582T | *Fraxinus chinensis* | China | MH121517 | MH121435 | – | MH121559 | – |
| *D. fructicola* | MAFF 246408T | *Passiflora edulis* | Japan | LC342734 | LC342738 | LC342737 | LC342735 | LC342736 |
| *D. fujianensis* | JZB 320149T | *Camellia sinensis* | China | MW010212 | MW205212 | – | MW20523 | MW056008 |
| *D. fukushii* | MAFF 625034 | *Pyrus pyrifolia* | Japan | JQ807469 | – | – | JQ807418 | – |
| *D. fulvicolor* | PSCG 051T | *Pyrus pyrifolia* | China | MK626859 | MK691132 | MK726163 | MK654806 | MK691236 |
| *D. fusicola* | CGMCC 3.17087T | *Lithocarpus glabra* | China | KF576281 | KF576233 | – | KF576256 | KF576305 |
| *D. fusiformis* | JZB 320156T | *Camellia sinensis* | China | MW010218 | MW205218 | – | MW205234 | MW056014 |
| *D. gammata* | ZJUE 0318 T | *Citrus reticulata* | China | OR160316 | OR178783 | OR178799 | OR178815 | OR178831 |
| *D. gammata* | ZJUE 0319T | *Citrus reticulata* | China | OR160317 | OR178784 | OR178800 | OR178816 | OR178832 |
| *D. gammata* | ZJUE 0401 | *Citrus reticulata* | China | OR160318 | OR178785 | OR178801 | OR178817 | OR178833 |
| ***D. gammata*** | **CFCC 70722** | ***Juglans regia* L.** | **China** | **PP862872** | – | **PP868588** | **PP868526** | **PP868557** |
| ***D. gammata*** | **CFCC 70723** | ***Juglans regia* L.** | **China** | **PP862874** | – | **PP868590** | **PP868528** | **PP868559** |
| ***D. gammata*** | **CFCC 70724** | ***Juglans regia* L.** | **China** | **PP862873** | – | **PP868589** | **PP868527** | **PP868558** |
| ***D. gammata*** | **CFCC 70725** | ***Juglans regia* L.** | **China** | **PP862870** | – | **PP868586** | **PP868524** | **PP868555** |
| ***D. gammata*** | **CFCC 70726** | ***Juglans regia* L.** | **China** | **PP862871** | – | **PP868587** | **PP868525** | **PP868556** |
| *D. ganjae* | CBS 180.91T | *Cannabis sativa* | USA | KC343112 | KC343354 | KC343596 | KC343838 | KC344080 |
| *D. ganzhouensis* | CFCC 53087T | Unknown | China | MK432665 | MK442985 | MK443010 | MK578139 | MK578065 |
| *D. gardeniae* | CBS 288.56 | *Gardenia florida* | .Italy | KC343113 | KC343355 | KC343597 | KC343839 | KC344081 |
| *D. garethjonesii* | MFLUCC 12-0542aT | Unknown | Thailand | KT459423 | KT459470 | – | KT459457 | KT459441 |
| *D. glabrae* | SCHM.3622T | *Bougainvilleae glabrae* | China | AY601918 | – | – | – | – |
| *D. globoostiolata* | MFLUCC 23-0025T | Unknown | Thailand | 0Q600200 | – | – | 0Q603503 | 0Q678280 |
| *D. gossiae* | BRIP 59730aT | *Sesbania sp.* | Australia | 0M918693 | – | – | 0M960602 | 0M960620 |
| *D. goulteri* | BRIP 55657aT | *Helianthus annuus* | Australia | KJ197290 | – | – | KJ197252 | KJ197270 |
| *D. grandifton* | SAUCC194.84T | *Heterostemma grandiflorum* | China | MT822612 | MT855691 | MT85558 | MT855924 | MT855809 |
| *D. griceae* | BRIP 67014aT | *Solanum melongena* | Australia | 0M918694 | – | – | 0M960603 | 0M960621 |
| *D. guangdongensis* | ZHKUCC 20-0014T | *Citrus grandis* | China | MT355684 | MT409314 | – | MT409338 | MT409292 |
| *D. guangxiensis* | JZB 320094T | *Vitis vinifera* | China | MK335772 | MK736727 | – | MK523566 | MK500168 |
| *D. guizhouensis* | GZAAS 20-0338T | Unknown | China | 0M060254 | OL961763 | – | 0L961761 | OL961762 |
| *D. gulyae* | BRIP 54025T | *Helianthus annuus* | Australia | JF431299 | – | – | JN645803 | KJ197271 |
| *D. guttulata* | CGMCC 3.20100T | Unknown | China | MT385950 | MW022470 | MW022491 | MT424685 | MT424705 |
| *D. hainanenesis* | HNCM049 | *Camellia oleifera* | China | OR647684 | | OR671936 | OR671944 | OR671952 |
| *D. hartii* | BRIP 60285eT | *Macroptilium lathyroides* | Australia | OR019754 | – | – | OR039644 | OR039651 |
| *D. hazenia* | BRIP 75103a | Unknown | Australia | OR122283 | – | – | OR135527 | OR135528 |
| *D. helianthi* | CBS 592.81T | *Helianthus annuus* | Serbia | KC343115 | JX197454 | KC343599 | KC343841 | KC344083 |
| *D. helicis* | CBS 138596T | *Hedera helix* | France | KJ210538 | KJ435043 | KJ420875 | KJ210559 | KJ420828 |
| *D. heliconiae* | SAUCC 194.77T | *Heliconia metallica* | China | MT822605 | MT855684 | MT855573 | MT855917 | MT855802 |
| *D. heterophyllae* | CBS143769T | *Acacia heterophylla* | France | MG600222 | MG600218 | MG600220 | MG600224 | MG600226 |
| *D. heterostemmatis* | SAUCC194.85T | *Heterostemma grandiflorum* | China | MT822613 | MT855692 | MT855581 | MT855925 | MT855810 |
| *D. hickoriae* | CBS 145.267T | *Carya glabra* | USA | KC343118 | KC343360 | KC343602 | KC343844 | KC344086 |
| *D. hispaniae* | CBS 143351T | *Vitis vinifera* | Spain | MG281123 | MG281820 | MG281471 | MG281644 | MG281296 |
| *D. hongkongensis* | CBS115448T | *Dichroa febrifuga* | China | KC343119 | KC343361 | KC343603 | KC343845 | KC344087 |
| *D. hordei* | CBS 481.92 | *Hordeum vulgare* | Norway | KC343120 | KC343362 | KC343604 | KC343846 | KC344088 |
| *D. howardiae* | BRIP 59697aT | *Agave sp.* | Australia | 0M918695 | – | – | OM960604 | 0M960622 |
| *D. hsinchuensis* | NTUPPMCC 18-153-1T | *Camellia sinensis* | China | MZ268409 | MZ268451 | MZ268493 | MZ268472 | MZ268430 |
| *D. huairouensis* | CFCC 56809 | *Corylus heterophylla* | China | – | ON157946 | ON157980 | ON158015 | ON158050 |
| *D. huairouensis* | CFCC 56810T | *Corylus heterophylla* | China | – | ON157944 | ON157979 | ON158014 | ON158049 |
| *D. huangshanensis* | CNUCC 201903T | *Camellia oleifera* | China | MN219730 | – | MN224558 | MN224678 | MN227011 |
| *D. hubeiensis* | JZB 320123T | *Vitis vinifere* | China | MK335809 | MK500235 | – | MK523570 | MK500148 |
| *D. humulicola* | CT2018-1T | *Humulus lupulus* | USA | MN152927 | MN180204 | MN180213 | MN180207 | – |
| *D. hunanensis* | HNZZ023T | *Camellia oleifera* | China | MZ509550 | MZ504680 | MZ504691 | MZ504702 | MZ504713 |
| *D. hungariae* | CBS 143353T | *Vitis vinifera* | Hungary | MG281126 | MG281823 | MG281474 | MG281647 | MG281299 |

| | | | | | | | | |
|---|---|---|---|---|---|---|---|---|
| *D. hyperici* | GUCC 414.4T | *Hypericum patulum* | China | OP581227 | | | OP688532 | OP688557 |
| *D. iberica* | CECT 21218T | *Festuca rubra subsp. pruinosa* | Spain | ON159902 | ON364028 | ON398819 | ON398841 | ON364049 |
| *D. ilicicola* | FPH 2015502T | *I. verticillata* × *I. serrata* | USA | MH171064 | – | MH171084 | – | MH171074 |
| *D. impulsa* | CBS114434T | *Sorbus aucuparia* | Sweden | KC343121 | KC343363 | KC343605 | KC343847 | KC344089 |
| *D. incompleta* | CGMCC 3.18288T | *Camellia sinensis* | China | KX986794 | KX999289 | KX999265 | KX999186 | KX999226 |
| *D. inconspicua* | CBS 133813T | *Maytenus ilicifolia* | Brazil | KC343123 | KC343365 | KC343607 | KC343849 | KC344091 |
| *D. infecunda* | CBS 133812T | *Schinus terebinthifolius* | Brazil | KC343126 | KC343368 | KC343610 | KC343852 | KC344094 |
| *D. infertilis* | CBS 230.52T | *Citrus sinensis* | Paramaribo | KC343052 | KC343294 | KC343536 | KC343778 | KC344020 |
| *D. irregularis* | CGMCC 3.20092 | Unknown | China | MT385951 | MT424721 | – | MT424686 | MT424706 |
| *D. isoberliniae* | CBS137981T | *Isoberlinia angolensis* | Zambia | KJ869133 | – | – | – | KJ869245 |
| *D. italiana* | MFLUCC 18-0090T | *Morus alba* L. | Italy | MH846237 | MH853690 | – | MH853686 | MH853688 |
| *D. jazanensis* | PPDU28RT | *Coffea arabica* | Saudi Arabia | OQ586408 | – | – | OR137582 | OR137586 |
| *D. jinxiu* | CGMCC3.20269T | *Prunus persica* cv. Zaofenghuang | China | MW477881 | MW480869 | MW480865 | MW480873 | MW480877 |
| *D. jishouensis* | ZJUE 0265 T | *Citrus unshiu* | China | OR160319 | OR178786 | OR178802 | OR178818 | OR178834 |
| *D. juglandia* | CBS121004T | *Juglans sp.* | USA | KC343134 | KC343376 | KC343618 | KC343860 | KC344102 |
| *D. juglandicola* | CFCC 51134T | *Juglans mandshurica* | China | MW477881 | KX024616 | – | KX024628 | KX024634 |
| *D. juglandigena* | GUCC 422.16T | *Juglans regia* L. | China | OP581229 | – | – | OP688534 | OP688559 |
| *D. juglandigena* | GUCC 422.161 | *Juglans regia* L. | China | OP581230 | – | – | OP688535 | OP688560 |
| *D. kadsurae* | CFCC 52586T | *Kadsura longipedunculata* | China | MH121521 | MH121439 | MH121479 | MH121563 | MH121600 |
| *D. kochmanii* | BRIP 54033T | *Helianthus annuus* | Australia | JF431295 | – | – | JN645809 | – |
| *D,kongi* | BRIP 54031T | *Helianthus annuus* | Australia | JF431301 | – | – | JN645797 | KJ197272 |
| *D. krabiensis* | MFLUCC 17-2481T | *Bruquiera* sp. | Unknown | MN047101 | – | – | MN433215 | MN431495 |
| *D. lenispora* | CGMCC 3.20101T | Unknown | China | MT385952 | MW022472 | MW022493 | MT424687 | MT424707 |
| *D. leptostromiformis* | CBS 558.93T | *Lupinus* sp. | Western Australia | KC343244 | KC343486 | KC343728 | KC343970 | KC344212 |
| *D. leucospermi* | CBS 111980T | *Leucospermum* sp. | Australia | JN712460 | KY435663 | KY435653 | KY435632 | KY435673 |
| *D. limonicola* | CBS142549T | *Citrus limon* | Malta | MF418422 | MF418256 | MF418342 | MF418501 | MF418582 |
| *D. liquidambaris* | SCHM 3621T | *Liquidambaris formosanae* | China | AY601919 | – | – | – | – |
| *D. litchicola* | BRIP 54900T | *Litchi chinensis* | Australia | JX862533 | – | – | JX862539 | KF170925 |
| *D. litchii* | SAUCC 194.22T | *Litchi chinensis* | China | MT822550 | MT855635 | MT855519 | MT855863 | MT855747 |
| *D. lithocarp* | CGMCC 3.15175 | *Lithocarpus glabra* | China | KC153104 | – | – | KC153095 | KF576311 |
| *D. litoricola* | MFLUCC 16-1195T | Unknown | UK | MF190139 | – | – | – | – |
| *D. longicolla* | FAU 599T | *Glycine max* | USA | KJ590728 | KJ612124 | KJ659188 | KJ590767 | KJ610883 |
| *D. longiconidialis* | ZHKUCC 22-0058T | *Morinda officinalis* | China | ON322887 | – | ON315017 | ON315045 | ON315076 |
| *D. longipapillata* | ZHKUCC 22-0180T | Unknown | China | OR164928 | OR166301 | – | OR166277 | OR166313 |
| *D. longipapillata* | ZHKUCC 22-0190 | Unknown | China | OR164929 | OR166302 | – | OR166278 | OR166314 |
| *D. longispora* | CBS 194.36T | *Ribes* sp. | Canada | KC343135 | KC343377 | KC343619 | KC343861 | KC344103 |
| *D. lonicerae* | MFLUCC 17-0963T | *Lonicera* sp. | Italy | KY964190 | KY964116 | – | KY964146 | KY964073 |
| *D. lovelaceae* | BRIP 60163aT | *Amaranthus blitum* | Australia | 0M918696 | – | – | 0M960605 | 0M960623 |
| *D. lusitanicae* | CBS 123212T | *Foeniculum vulgare* | Portugal | KC343136 | KC343378 | KC343620 | KC343862 | KC344104 |
| *D. lutescens* | SAUCC194.36T | *Chrysalidocarpus lutescens* | China | MT822564 | MT855647 | MT855533 | MT855877 | MT855761 |
| *D. macadamiae* | BRIP 66526T | *Macadamia* sp. | Australia | MN708230 | – | – | MN696528 | MN696539 |
| *D. machili* | SAUCC 194.111T | *Machilus pingi* | China | MT822639 | MT855718 | MT855606 | MT855951 | MT855836 |
| *D. macintoshii* | BRIP 55064aT | *Rapistrum rugosum* | Australia | KJ197289 | – | – | KJ197251 | KJ197269 |
| *D. malorum* | CBS142383T | *Malus domestica* | Portugal | KY435638 | KY435658 | KY435648 | KY435627 | KY435668 |
| *D. manihotia* | CBS 505.76 | *Manihot utilissima* | Rawanda | KC343138 | KC343380 | KC343622 | KC343864 | KC344106 |
| *D marina* | MFLU 17-2622T | *Rhizophora* sp. | Thailand | MN047102 | – | – | – | – |
| *D. maritima* | DAOMC 250563T | *Picea rubens needle* | Canada | KU552025 | – | – | KU552023 | KU574615 |
| *D. masirevicii* | BRIP 57892aT | *Helianthus annuus* | Australia | KJ197277 | – | – | KJ197239 | KJ197257 |
| *D. mayteni* | CBS 133185T | *Maytenus ilicifolia* | Brazil | KC343139 | KC343381 | KC343623 | KC343865 | KC344107 |
| *D. maytenicola* | CBS136441T | *Maytenus acuminata var. acuminata* | South Africa | KF777157 | – | – | – | KF777250 |
| *D. mclennaniae* | BRIP 60072aT | *Mangifera laurina* | Australia | 0M918697 | – | – | 0M960606 | 0M960624 |
| *D. mediterranea* | DAL-34T | *Prunus persica* L. | Spain | MT007489 | MT006761 | MT007095 | MT006989 | MT006686 |
| *D. megabiguttulata* | ZHKUCC 22-0067T | *Morinda officinalis* | China | ON322895 | ON315001 | ON315025 | – | ON315085 |
| *D. megalospora* | CBS 143.27T | *Sambucus canadensis* | Unknown | KC343140 | KC343382 | KC343624 | KC343866 | KC344108 |
| *D. melastomatis* | SAUCC 194.55T | *Melastoma malabathricum* | China | MT822583 | MT855664 | MT855551 | MT855896 | MT855780 |

| | | | | | | | | |
|---|---|---|---|---|---|---|---|---|
| *D meliae* | CFCC 53089T | *Melia azedarach* | China | MK432657 | – | 0N081662 | ON081654 | MK578057 |
| *D. melitensis* | CBS142551T | *Citrus limon* | Malta | MF418424 | MF418258 | MF418344 | MF418503 | MF418584 |
| *D. melongenae* | MBELPIC61.1T | *Solanum melongena* | Pulo | OQ123525 | – | – | OR099713 | OR099714 |
| *D. melonis* | CBS 507.78T | *Cucumis melo* | USA | KC343142 | KC343384 | KC343626 | KC343868 | KC344110 |
| *D. micheliae* | SCHM 3603T | *Micheliae albae* DC. | China | AY620820 | – | – | – | – |
| *D. middletonii* | BRIP 54884eT | *Rapistrum rugosum* | Australia | KJ197286 | – | – | KJ197248 | KJ197266 |
| *D. millettiae* | GUCC 9167T | *Millettia reticulata* | China | MK398674 | MK502086 | – | MK480609 | MK502089 |
| *D. minima* | CGMCC 3.20097T | Unknown | China | MT385953 | MT424722 | MW022496 | MT424688 | MT424708 |
| *D. minusculata* | CGMCC 3.20098T | Unknown | China | MT385957 | MW022475 | MW022499 | MT424692 | MT424712 |
| *D. miriciae* | BRIP 54736jT | *Helianthus annuus* | Australia | KJ197283 | – | – | KJ197245 | KJ197263 |
| *D. monetii* | MF-Ha18-049T | *Helianthus annuus* | Russia | MW008494 | MZ671939 | MZ671965 | MW008516 | MW008505 |
| *D. moorei* | BRIP 61500bT | *Vigna radiata* | Australia | OR019755 | – | – | OR039645 | OR039652 |
| *D. morindae* | ZHKUCC 22-0072 | *Morinda officinalis* | China | ON322900 | ON315003 | ON315030 | ON315057 | ON315090 |
| *D. morindendophytica* | ZHKUCC 22-0069T | *Morinda officinalis* | China | ON322897 | | ON315027 | ON315054 | ON315087 |
| *D. moriniae* | BRIP 60190aT | *Xanthium strumarium* | Australia | 0M918698 | – | – | 0M960607 | 0M960625 |
| *D. multigutullata* | ICMP 20656T | *Citrus grandis* | China | KJ490633 | – | KJ490575 | KJ490512 | KJ490454 |
| *D. musigena* | CBS 129519T | *Musa* sp. | Australia | KC343143 | KC343385 | KC343627 | KC343869 | KC344111 |
| *D. myracrodruonis* | URM 7972T | *Myracrodruon urundeuva* | Unknown | MK205289 | MK205290 | – | MK213408 | MK205291 |
| *D. nanjingensis* | CFCC 55348T | *Acer palmatum* | China | MZ560721 | MZ577308 | MZ577299 | MZ577281 | MZ577290 |
| *D. neatei* | BRIP 60289aT | *Bauhinia sp.* | Australia | OR019756 | – | – | OR039646 | OR039653 |
| *D. nebulae* | PMM 1681T | *Vitis vinifera* | South Africa | KY511337 | – | – | MH708552 | KY511369 |
| *D. neilliae* | CBS 144.27T | *Spiraea* sp. | USA | KC343144 | KC343386 | KC343628 | KC343870 | KC344112 |
| *D. neoarctii* | CBS109490 | *Ambrosia trifida* | USA | KC343145 | KC343387 | KC343629 | KC343871 | KC344113 |
| *D. neoraonikayaporum* | MFLUCC 14-1136T | *Tectona grandis* | Thailand | KU712449 | KU749356 | – | KU749369 | KU743988 |
| *D. nigra* | JZB 320170T | *Ballota nigra* L. | Italy | MN653009 | – | – | MN892277 | MN887113 |
| *D. nobilis* | CBS 587.79T | *Pinus parviflora* | Japan | KC343153 | KC343395 | KC343637 | KC343879 | KC344121 |
| *D. nomurai* | CBS 157.29T | *Morus sp.* | Japan | KC343154 | KC343396 | KC343638 | KC343880 | KC344122 |
| *D. norfolkensis* | BRIP 59718aT | *Mangifera indica* | Australia | 0M918699 | – | – | 0M960608 | 0M960626 |
| *D. nothofagi* | BRIP 54801T | *Nothofagus cunninghamii* | Australia | JX862530 | – | – | JX862536 | KF170922 |
| *D. novem* | CBS 127271T | *Maytenus ilicifolia* | Brazil | KC343157 | KC343399 | KC343641 | KC343883 | KC344125 |
| *D. novem* | CBS 117165 | *Glycine max* L. | Serbia | KC343156 | KC343398 | KC343640 | KC343882 | KC344124 |
| *D. obtusifoliae* | CBS143449T | *Acacia obtusifolia* | Australia | MG386072 | – | MG386137 | – | – |
| *D. ocoteae* | CBS 141330T | *Ocotea obtusata* | France | KX228293 | – | – | – | KX228388 |
| *D. oculi* | HHUF 30565T | Unknown | Japan | LC373514 | – | – | LC373516 | LC373518 |
| ***D. olivacea*** | **CFCC 70713T** | ***Juglans regia* L.** | **China** | **PP862887** | **PP868515** | **PP868603** | **PP868541** | **PP868572** |
| ***D. olivacea*** | **CFCC 70714** | ***Juglans regia* L.** | **China** | **PP862886** | **PP868514** | **PP868602** | **PP868540** | **PP868571** |
| ***D. olivacea*** | **CFCC 70715** | ***Juglans regia* L.** | **China** | **PP862885** | **PP868513** | **PP868601** | **PP868539** | **PP868570** |
| ***D. olivacea*** | **CFCC 70716** | ***Juglans regia* L.** | **China** | **PP862888** | **PP868516** | **PP868604** | **PP868542** | **PP868573** |
| ***D. olivacea*** | **CFCC 70717** | ***Juglans regia* L.** | **China** | **PP862889** | **PP868517** | **PP868605** | **PP868543** | **PP868574** |
| *D oncostoma* | CBS 589.78T | *Robinia pseudoacacia* | Germany | KC343162 | KC343404 | KC343646 | KC343888 | KC344130 |
| *D. oraccinii* | LC 3166T | *Camellia sinensis* | China | KP267863 | – | KP293517 | KP267937 | KP293443 |
| *D. orixae* | HKAS 121465T | *Orixa japonica* | China | OK283041 | OK484485 | 0K484486 | OK432279 | 0K432278 |
| *D. osmanthi* | GUCC 9165T | *Litchi chinensis* | China | MK398675 | MK502087 | – | MK480610 | MK502090 |
| *D. ovalispora* | ICMP 20659T | *Citrus limon* | China | KJ490628 | – | KJ490570 | KJ490507 | KJ490449 |
| *D. ovoidea* | CGMCC 3.17092T | *Lithocarpus glabra* | China | KF576264 | KF576222 | – | KF576239 | KF576288 |
| *D. oxe* | CBS 133186T | *Maytenus ilicifolia* | Brazil | KC343164 | KC343406 | KC343648 | KC343890 | KC344132 |
| *D. pachirae* | COAD 2074T | *Pachira glabra* | Brazil | MG559537 | MG559535 | – | MG559539 | MG559541 |
| *D. padi var.padi* | CBS 114200T | *Prunus padus* | Sweden | KC343169 | KC343411 | KC343653 | KC343895 | KC344137 |
| *D. padina* | CFCC 52590T | *Padus racemosa* | China | MH121525 | MH121443 | MH121483 | MH121567 | MH121604 |
| *D. pandanicola* | MFLUCC 17-0607T | *Pandanaceae* | Thailand | MG646974 | – | – | – | MG646930 |
| *D. paranensis* | CBS133184T | *Maytenus ilicifolia* | Brazil | KC343171 | KC343413 | KC343655 | KC343897 | KC344139 |
| *D. parapterocarpi* | CBS 137986T | *Pterocarpus brenanii* | Zambia | KJ869138 | – | – | – | KJ869248 |
| *D. parva* | PSCG 034T | *Pyrus bretschneideri* | China | MK626919 | – | MK726210 | MK654858 | MK691248 |
| *D. pascoei* | BRIP 54847T | *Persea americana* | Australia | JX862532 | – | – | JX862538 | KF170924 |
| *D. passiflorae* | CBS132527T | *Passiflora edulis* | South Africa | JX069860 | KY435664 | KY435654 | KY435633 | KY435674 |
| *D. passifloricola* | CBS 141329T | *Passiflora foetida* | Malaysia | KX228292 | – | KX228367 | – | KX228387 |
| *D. patagonica* | CBS145291T | *Aristotelia chilensis* | Chile | MN509717 | MN974279 | – | MN509739 | MN509728 |
| *D. penetriteum* | LC 3353T | *Camellia sinensis* | China | KP714505 | – | KP714493 | KP714517 | KP714529 |
| *D. perjuncta* | CBS 109745T | *Ulmus glabra* | Austria | KC343172 | KC343414 | KC343656 | KC343898 | KC344140 |
| *D. perniciosa* | CBS124030T | *Malus pumila* | New Zealand | KC343149 | KC343391 | KC343633 | KC343875 | KC344117 |
| *D. perseae* | CBS 151.73T | *Persea gratissima* | Netherlands | KC343173 | KC343415 | KC343657 | KC343899 | KC344141 |
| *D. pescicola* | MFLUCC 16-0105T | *Prunus persica* | China | KU557555 | KU557603 | – | KU557623 | KU557579 |

| | | | | | | | | |
|---|---|---|---|---|---|---|---|---|
| *D. phaseolorum* | AR4203T | *Phaseolus vulgaris* | USA | KJ590738 | KJ612135 | KJ659220 | KJ590739 | KJ610893 |
| *D. phillipsi* | CAA 817T | *Vaccinium corymbosum* | Portugal | MK792305 | MK883831 | MK871445 | MK828076 | MN000351 |
| *D. phragmitis* | CBS 138897T | *Phragmites australis* | China | KP004445 | – | KP004503 | – | KP004507 |
| *D. phyllanthicola* | SCHM 3680T | *Phyllanth i emblicae* L. | China | AY620819 | – | – | – | – |
| *D. platzii* | BRIP 60353aT | *Persea americana* | Austria | 0M918700 | – | – | 0M960609 | 0M960627 |
| *D. podocarpi-macrophylli* | CGMCC 3.18281T | *Podocarpus macrophyllus* | Japan | KX986774 | KX999278 | KX999246 | KX999167 | KX999207 |
| *D. poincianellae* | URM 7932T | *Poincianella pyramidalis* | Brazil | MH989509 | MH989540 | MH989539 | MH989538 | MH989537 |
| *D. pometiae* | SAUCC 194.72T | *Pometia pinnata* | China | MT822600 | MT855679 | MT855568 | MT855912 | MT855797 |
| *D. portugallica* | CBS 144228T | *Camellia sinensis* | Portugal | MH063905 | MH063893 | MH063899 | MH063911 | MH063917 |
| *D. pseudoanacardii* | CBS 148909T | *Trema guineensis* | Cameroon | OR348655 | OR468831 | OR468801 | OR468811 | OR468821 |
| | STMA 18247 | *Trema guineensis* | Cameroon | 0R348656 | OR468830 | OR468800 | OR468810 | OR468820 |
| | STMA 18292 | *Trema guineensis* | Cameroon | OR348657 | OR468829 | OR468799 | OR468809 | OR468819 |
| *D. pseudoalnea* | CFCC 54190T | *Alnus glutinosa* | Netherlands | MZ727037 | MZ753468 | MZ781302 | MZ816343 | MZ753487 |
| *D. pseudobiguttulata* | CMP 20657T | *Citrus limon* | China | KJ490582 | – | KJ490524 | KJ490461 | KJ490403 |
| *D. pseudofoliicola* | HNCM045 | *Camellia oleifera* | China | OR647680 | | OR671932 | OR671940 | OR671948 |
| *D. pseudoinconspicua* | URM 7874T | *Poincianella pyramidalis* | Brazil | MH122538 | MH122528 | MH122517 | MH122533 | MH122524 |
| *D. pseudomangiferae* | CBS 101339T | *Mangifera indica* | Dominican Republic | KC343181 | KC343423 | KC343665 | KC343907 | KC344149 |
| *D. pseudooculi* | HHUF 30617T | Unknown | Japan | LC373515 | – | – | LC373517 | LC373519 |
| *D. pseudophoenicicola* | CBS 462.69T | *Phoenix dactylifera* | Spain | KC343184 | KC343426 | KC343668 | KC343910 | KC344152 |
| *D. pseudotsugae* | MFLU 15-3228T | *Pseudotsuga menziesii* | Italy | KY964225 | KY964138 | – | KY964181 | KY964108 |
| *D. psoraleae* | CBS 136412T | *Psoralea pinnata* | South Africa | KF777158 | – | – | KF777245 | KF777251 |
| *D. psoraleae-pinnatae* | CBS 136413T | *Psoralea pinnata* | South Africa | KF777159 | – | – | – | KF777252 |
| *D. pterocarpi* | MFLUCC10-0571 | *Pterocarpus indicus* | Thailand | JQ619899 | JX197451 | – | JX275416 | JX275460 |
| *D. pterocarpicola* | MFLUCC 10-0580a | *Pterocarpus indicus* | Thailand | JQ619887 | JX197433 | – | JX275403 | JX275441 |
| *D. pulla* | CBS 338.89T | *Hedera helix* | Yugoslavia | KC343152 | KC343394 | KC343636 | KC343878 | KC344120 |
| *D. pungensis* | SAUCC 194.112T | *Elaeagnus pungens* | China | MT822640 | MT855719 | MT855607 | MT855952 | MT855837 |
| *D. pustulata* | CBS 109742 | *Acer pseudoplatanus* | Austria | KC343185 | KC343427 | KC343669 | KC343911 | KC344153 |
| *D. pyracanthae* | CBS142384T | *Pyracantha coccinea* | Portugal | KY435635 | KY435656 | KY435645 | KY435625 | KY435666 |
| *D. quercicola* | CSUFTCC 104T | *Quercus aliena* | China | ON076567 | ON081670 | ON081667 | 0N081659 | – |
| *D. racemosae* | CBS143770T | *Euclea racemosa* | South Africa | MG600223 | MG600219 | MG600221 | MG600225 | MG600227 |
| *D. raonikayaporum* | CBS 133182T | *Spondias mombin* | Brazil | KC343188 | KC343430 | KC343672 | KC343914 | KC344156 |
| *D. rauvolfiae* | CBS 148912T | *Rauvolfia vomitoria* | Cameroon | 0R348658 | OR468828 | OR468798 | OR468808 | OR468818 |
| *D. ravennica* | MFLUCC 15-0479T | *Clematis vitalba* | Italy | KU900335 | – | – | KX365197 | KX432254 |
| *D. rhodomyrt* | CFCC 53101T | *Rhodomyrtus tomentosa* | China | MK432643 | MK442965 | MK442990 | MK578119 | MK578046 |
| *D. rhoina* | CBS 146.27 | *Rhus toxicodendron* | Unknown | KC343189 | KC343431 | KC343673 | KC343915 | KC344157 |
| *D. rizhaoensis* | CFCC 57562T | *Xanthium strumarium* | China | OP955930 | OP959782 | OP959785 | OP959767 | OP959773 |
| *D. rosae* | MFLUCC 17-2658T | *Rosa* sp. | UK | MG828894 | MG829273 | – | – | MG843878 |
| *D. rosicola* | MFLU 17-0646T | *Rosa* sp. | UK | MG828895 | MG829274 | – | MG829270 | MG843877 |
| *D. rosiphthora* | COAD 2913T | *Rosa* sp. | Brazil | MT311196 | MT313690 | – | MT313692 | – |
| *D. rossmaniae* | CAA 762T | *Vaccinium corymbosum* | Portugal | MK792290 | MK883822 | MK871432 | MK828063 | MK837914 |
| *D. rostrata* | CFCC 50062T | *Juglans mandshurica* | China | KP208847 | KP208849 | KP208851 | KP208853 | KP208855 |
| *D. rudis* | CBS113201 | *Clematis serratifolia* | Belgium | KC343234 | KC343476 | KC343718 | KC343960 | KC344202 |
| *D. ruiliensis* | ZJUE 0307 T | *Citrus limon* | China | OR160315 | OR178782 | OR178798 | OR178814 | OR178830 |
| *D. rumicicola* | MFLUCC 18-0739T | *Rumex* sp. | Italy | MH84623 | – | – | MK049554 | MK049555 |
| *D. saccarata* | CBS116311T | *Protea repens* | South Africa | KC343190 | KC343432 | KC343674 | KC343916 | KC344158 |
| *D. sackstonii* | BRIP 54669bT | *Helianthus annuus* | Australia | KJ197287 | – | – | KJ197249 | KJ197267 |
| *D. salicicola* | BRIP 54825T | *Salix purpuree* | Australia | JX862531 | – | – | JX862537 | KF170923 |
| *D. salinicola* | MFLU 18-0553T | *Xylocarpus* sp. | Thailand | MN047098 | – | – | MN077073 | – |
| *D. samaneae* | SDBR-CMU470T | *Samanea saman* (Jacq.) Merr. | Thailand | 0Q600197 | 0Q646884 | 0Q646880 | 0Q603500 | 0Q678277 |
| *D. sambuci* | CFCC 51986T | *Sambucus williamsii* | China | KY852495 | KY852499 | KY852503 | KY852507 | KY852511 |
| *D. sapindicola* | CFCC 55344T | *Sapindus mukorossi* | China | MW881507 | MW898943 | MW898940 | MW898934 | MW898937 |
| *D. schimae* | CFCC 53103T | *Schima superbe* | China | MK432640 | MK442962 | MK442987 | MK578116 | MK578043 |
| *D. schini* | CBS133181T | *Schinus terebinthifolius* | Brazil | KC343191 | KC343433 | KC343675 | KC343917 | KC344159 |
| *D. schisandrae* | CFCC 51988T | *Schisandra chinensis* | China | KY852497 | KY852501 | KY852505 | KY852509 | KY852513 |
| *D. schoeni* | MFLU 15-1279T | *Schoenus nigricans* | Italy | KY964226 | KY964139 | – | KY964182 | KY964109 |
| *D. sclerotioides* | CBS 296.67T | *Cucumis sativus* | Netherlands | KC343193 | KC343435 | KC343677 | KC343919 | KC344161 |

| | | | | | | | | |
|---|---|---|---|---|---|---|---|---|---|
| *D. scobina* | CBS 251.38T | *Fraxinus excelsior* | UK | KC343195 | KC343437 | KC343679 | KC343921 | KC344163 |
| *D. searlei* | BRIP 66528T | *Macadamia* sp. | Australia | MN708231 | – | – | – | MN696540 |
| *D. sennae* | CFCC 51636T | *Senna bicapsularis* | China | KY203724 | KY228875 | – | KY228885 | KY228891 |
| *D. sennicola* | CFCC 51634T | *Senna bicapsularis* | China | KY203722 | KY228873 | – | KY228883 | KY228889 |
| *D. serafiniae* | BRIP 55665aT | *Helianthus annuus* | Australia | KJ197274 | – | – | KJ197236 | KJ197254 |
| *D. sexualispora* | ZJUE 0418 T | *Citrus unshiu* | China | OR160330 | OR178797 | OR178813 | OR178829 | OR178845 |
| *D shaanxiensis* | CFCC 53106T | *Ljana* sp. | China | MK432654 | MK442976 | MK443001 | MK578130 | – |
| ***D. shangluoensis*** | **CFCC 70727** | ***Juglans regia* L.** | **China** | **PP862877** | **PP868505** | **PP868593** | **PP868531** | **PP868562** |
| ***D. shangluoensis*** | **CFCC 70728T** | ***Juglans regia* L.** | **China** | **PP862875** | **PP868503** | **PP868591** | **PP868529** | **PP868560** |
| ***D. shangluoensis*** | **CFCC 70729** | ***Juglans regia* L.** | **China** | **PP862878** | **PP868506** | **PP868594** | **PP868532** | **PP868563** |
| ***D. shangluoensis*** | **CFCC 70730** | ***Juglans regia* L.** | **China** | **PP862876** | **PP868504** | **PP868592** | **PP868530** | **PP868561** |
| ***D. shangluoensis*** | **CFCC 70731** | ***Juglans regia* L.** | **China** | **PP862879** | **PP868507** | **PP868595** | **PP868533** | **PP868564** |
| ***D. shangluoensis*** | **CFCC 70732** | ***Juglans regia* L.** | **China** | **PP862880** | **PP868508** | **PP868596** | **PP868534** | **PP868565** |
| ***D. shangrilaensis*** | **CFCC 70703T** | ***Juglans regia* L.** | **China** | **PP862896** | **PP868519** | **PP868612** | **PP868550** | **PP868581** |
| ***D. shangrilaensis*** | **CFCC 70704** | ***Juglans regia* L.** | **China** | **PP862897** | **PP868520** | **PP868613** | **PP868551** | **PP868582** |
| ***D. shangrilaensis*** | **CFCC 70705** | ***Juglans regia* L.** | **China** | **PP862898** | **PP868521** | **PP868614** | **PP868552** | **PP868583** |
| ***D. shangrilaensis*** | **CFCC 70706** | ***Juglans regia* L.** | **China** | **PP862899** | **PP868522** | **PP868615** | **PP868553** | **PP868584** |
| ***D. shangrilaensis*** | **CFCC 70707** | ***Juglans regia* L.** | **China** | **PP862900** | **PP868523** | **PP868616** | **PP868554** | **PP868585** |
| ***D. shangrilaensis*** | **CFCC 70708** | ***Juglans regia* L.** | **China** | **PP862895** | **PP868518** | **PP868611** | **PP868549** | **PP868580** |
| *D shawiae* | BRIP 64534aT | *Chloris gayana* | Australia | 0M918701 | – | – | OM960610 | 0M960628 |
| *D. shennongjiaensis* | CNUCC201905T | *Juglans regia* L. | China | MN216229 | MN224551 | MN224559 | MN224672 | MN227012 |
| *D. siamensis* | MFLUCC 10-0573a | *Dasymaschalon* sp. | Thailand | J0619879 | – | – | JX275393 | JX275429 |
| *D. silvicola* | CFCC 54191T | *Fraxinus excelsior* | Netherlands | MZ727041 | MZ753472 | MZ753481 | MZ816347 | MZ753491 |
| *D. sinensis* | CGMCC 3.19521T | *Amaranthus* sp. | China | MK637451 | – | – | MK660449 | MK660447 |
| *D. smilacicola* | CFCC 54582T | *Smilax glabra* | China | OP955933 | OP959779 | OP959788 | OP959770 | OP959776 |
| *D. soiae* | CBS 139282T | *Glycine max* | USA | KJ590719 | KJ612116 | KJ659208 | KJ590762 | KJ610875 |
| *D. solani-melongenae* | MCC-MNH 2729 | *Solanum melongena* | Philippines | OQ123551 | | | OR943642 | OR943679 |
| *D. spartinicola* | CBS140003T | *Spartium junceum* | Spain | KR611879 | – | KR857696 | – | KR857695 |
| *D. spinosa* | PSCG 383T | *Pyrus pyrifolia* | China | MK626849 | MK691129 | MK726156 | MK654811 | MK691234 |
| *D. sterilis* | CBS 136969T | *Vaccinium corymbosum* | ltaly | KJ160579 | KJ160548 | MF418350 | KJ160611 | KJ160528 |
| *D. stewartii* | CBS 193.36 | *Cosmos bipinnatus* | USA | FJ889448 | – | – | GQ250324 | – |
| *D stictica* | CBS 370.54 | *Buxus sampervirens* | Italy | KC343212 | KC343454 | KC343696 | KC343938 | KC344180 |
| *D. subclavata* | ICMP 20663T | *Citrus unshiu* | China | KJ490630 | – | KJ490572 | KJ490509 | KJ490451 |
| *D. subcylindrospora* | KUMCC 17-0151T | Unknown | China | MG746629 | – | – | MG746630 | MG746631 |
| *D. subellipicola* | KUMCC 17-0153T | Unknown | China | MG746632 | – | – | MG746633 | MG746634 |
| *D. subordinaria* | CBS101711T | *Plantago lanceolata* | New Zealand | KC343213 | KC343455 | KC343697 | KC343939 | KC344181 |
| *D. talong* | MCC-MNH 2727T | *Solanum melongena* | Philippines | OQ123545 | – | – | OR943637 | OR943674 |
| *D. taoicola* | MFLUCC 16-0117T | *Prunus persica* | China | KU557567 | – | – | KU557635 | KU557591 |
| *D. tarchonanthi* | CBS146073T | *Tarchonanthus littoralis* | South Africe | MT223794 | – | MT223759 | – | MT223733 |
| *D. tecomae* | CBS 100547T | *Tabebuia* sp. | Brazil | KC343215 | KC343457 | KC343699 | KC343941 | KC344183 |
| *D tectonae* | MFLUCC 12-0777T | *Tectona grandis* | Thailand | KU712430 | KU749345 | – | KU749359 | KU743977 |
| *D. tectonendophytica* | MFLUCC 13-0471T | *Tectona grandis* | Thailand | KU712439 | KU749354 | – | KU749367 | KU743986 |
| *D. tectonigena* | MFLUCC 12-0767T | *Camellia sinensis* | China | KU712429 | KU749358 | – | KU749371 | KU743976 |
| *D. terebinthifolii* | CBS 133180T | *Schinus terebinthifolius* | Brazil | KC343216 | KC343458 | KC343700 | KC343942 | KC344184 |
| *D. thunbergiae* | MFLUCC 10-0756aT | *Thunbergia laurifolia* | Thailand | JQ619893 | JX197440 | – | JX275409 | JX275449 |
| *D. thunbergiicola* | MFLUCC 12-0033T | *Thunbergia laurifolia* | Thailand | KP715097 | – | – | KP715098 | – |
| *D. tibetensis* | CFCC 51999T | *Juglandis regia* | China | MF279843 | MF279888 | MF279828 | MF279858 | MF279873 |
| ***D. tibetensis*** | **CFCC 70702** | ***Juglans regia* L.** | **China** | **PP862890** | – | **PP868606** | **PP868544** | **PP868575** |
| ***D. tibetensis*** | **CFCC 70709** | ***Juglans regia* L.** | **China** | **PP862891** | – | **PP868607** | **PP868545** | **PP868576** |
| ***D. tibetensis*** | **CFCC 70710** | ***Juglans regia* L.** | **China** | **PP862892** | – | **PP868608** | **PP868546** | **PP868577** |
| ***D. tibetensis*** | **CFCC 70711** | ***Juglans regia* L.** | **China** | **PP862893** | – | **PP868609** | **PP868547** | **PP868578** |
| ***D. tibetensis*** | **CFCC 70712** | ***Juglans regia* L.** | **China** | **PP862894** | – | **PP868610** | **PP868548** | **PP868579** |
| *D. tongrenensis* | GUCC 421.101 | *Juglans regia* L. | China | OP581226 | – | – | OP688531 | OP688556 |
| *D. tongrenensis* | GUCC 421.10T | *Juglans regia* L. | China | OP581225 | – | – | OP688530 | OP688555 |
| *D. torilicola* | MFLUCC 17-1051T | *Torilis arvensis* | ltaly | KY964212 | KY964127 | – | KY964168 | KY964096 |
| *D. toxica* | CBS 534.93T | *Lupinus angustifolius* | Australia | KC343220 | KC343462 | KC343704 | KC343946 | KC344188 |
| *D. toxicodendri* | FFPRI 420987T | *Toxicodendron vernicifluum* | Japan | LC275192 | LC275200 | LC275216 | LC275216 | LC275224 |
| *D treyorrowi* | BRIP 70737aT | *Cucumis melo* | Australia | 0M918703 | – | – | 0M960612 | 0M960630 |
| *D. tulliensis* | BRIP 62248a | *Theobroma cacao* | Australia | KR936130 | – | – | KR936133 | KR936132 |
| *D. tuyouyouiae* | BRIP 75017aT | *Decalobanthus peltatus* | Australia | 0Q917074 | – | – | 0Q889558 | 0Q889559 |

| | | | | | | | | |
|---|---|---|---|---|---|---|---|---|
| *D. ueckeri* | FAU 656T | *Cucumis melo* | USA | KJ590726 | KJ612122 | KJ659215 | KJ590747 | KJ610881 |
| *D. ukurunduensis* | CFCC 52592T | *Acer ukurunduense* | China | MH121527 | MH121445 | MH121485 | MH121569 | – |
| *D. ulmina* | CFCC 58828T | *Ulmus pumila* | China | OQ912957 | OQ910232 | OQ910262 | OQ910290 | OQ910324 |
| *D. ulmina* | CFCC 58829 | *Ulmus pumila* | China | OQ912958 | OQ910233 | OQ910263 | OQ910291 | OQ910325 |
| *D. ulmina* | CFCC 58830 | *Ulmus pumila* | China | OQ912959 | – | – | – | – |
| *D. undulata* | CGMCC 3.18293T | Unknown | China | KX986798 | – | KX999269 | KX999190 | KX999230 |
| *D. unshiuensis* | CGMCC3.17569T | *Citrus unshiu* | China | KJ490587 | – | KJ490529 | KJ490466 | KJ490408 |
| *D. vaccinii* | CBS 160.32T | *Oxycoccus macrocarpos* | USA | AF317578 | KC343470 | KC343712 | GQ250326 | KC344196 |
| *D. vacuae* | CAA 830T | *Vaccinium corymbosum* | Portugal | MK792309 | MK883834 | MK871449 | MK828080 | MK837931 |
| *D. vangoghii* | MF-Ha18-046T | *Helianthus annuus* | Russia | MW008492 | MZ671937 | MZ671963 | MW008514 | MW008503 |
| *D. vangueriae* | CBS137985T | *Vangueria infausta* | Zambia | KJ869137 | – | – | – | KJ869247 |
| *D. vawdrey* | BRIP 57887a | *Psidium guajava* | Australia | KR936126 | – | – | KR936129 | KR936128 |
| *D. velutina* | CGMCC 3.18286T | *Neolitsea* sp. | China | KX986790 | – | KX999261 | KX999182 | KX999223 |
| *D. vemiciicola* | CFCC 53109T | *Vernicia montana* | China | MK573944 | MK574583 | MK574599 | MK574619 | MK574639 |
| *D. vexans* | CBS127.14 | *Solanum melongena* | USA | KC343229 | KC343471 | KC343713 | KC343955 | KC344197 |
| *D. viciae* | JZB 320179T | *Vicia villosa* | China | OP626092 | – | OP627279 | OP627280 | OP627281 |
| *D. viniferae* | JZB 320071T | *Vitis vinifera* | China | MK341551 | MK500119 | – | MK500107 | MK500112 |
| *D. virgiliae* | CBS138788T | *Virgilia oroboides* | South Africa | KP247573 | – | – | – | KP247582 |
| *D. vitimegaspora* | STE-U 2675 | *Vitis vinifera* | China | AF230749 | – | – | – | – |
| *D. vochysiae* | LGMF 1583T | *Vochysia divergens* | Brazil | MG976391 | MK007528 | MK033323 | MK007526 | MK007527 |
| *D. woolworthii* | CBS 148.27 | *Ulmus americana* | Unknown | KC343245 | KC343487 | KC343729 | KC343971 | KC344213 |
| *D. xishuangbanica* | CGMCC 3.18282T | *Camellia sinensis* | China | KX986783 | – | KX999255 | KX999175 | KX999216 |
| *D. xunwuensis* | CFCC 53085T | Unknown | China | MK432663 | MK442983 | MK443008 | MK578137 | MK578063 |
| *D. yunnanensis* | CGMCC 3.18289T | *Coffea* sp. | China | KX986796 | KX999290 | KX999267 | KX999188 | KX999228 |
| *D. zaobaisu* | PSCG 031T | *Pyrus bretschneideri* | China | MK626922 | – | MK726207 | MK654855 | MK691245 |
| *D. zaofenghuang* | CGMCC3.20271T | *Prunus persica cv. Zaofenghuang* | China | MW477883 | MW480867 | – | MW480871 | MW480875 |
| *D. zhaoqingensis* | ZHKUCC 22-0056T | *Morinda officinalis* | China | ON322885 | ON315000 | ON315015 | – | ON315074 |
| *Diaporthella corylina* | CBS121124 | *Corylus* sp. | NA | KC343004 | KC343246 | KC343488 | KC343730 | KC343972 |

1 Acronyms: ATCC: American Type Culture Collection, Virginia, USA; BBH: BIOTEC Bangkok Herbarium, National Science and Technology Development Agency, Thailand; CBS: Westerdijk Fungal Biodiversity Institute (CBS-KNAW Fungal Biodiversity Centre), Utrecht, The Netherlands; CFCC: China Forestry Culture Collection Centre, Beijing, China; CMW: Culture Collection of Michael Wingfield, University of Pretoria, South Africa; CPC: Culture Collection of Pedro Crous, The Netherlands; IMI: Culture Collection of the International Mycological Institute, CABI Bioscience, Egham, Surrey, UK; MFLU: Mae Fah Luang University herbarium, Thailand; MFLUCC: Mae Fah Luang University Culture Collection, Thailand; MUCC: Murdoch University Culture Collection, Perth, Australia; NE: Gerard Adams Collections, University of Nebraska, Lincoln NE, USA; PPRI: Culture Collection of the Plant Protection Research Institute, Agriculture Research Center, Pretoria, South Africa; XJAU: Xinjiang Agricultural University, Xinjiang, China; NA: not applicable. All the new isolates used in this study are in bold and the type materials are marked with T.
