# Supplementary material for: Diversity and Pathogenicity of Six Diaporthe Species from Juglans regia in China
Source: J Fungi (Basel). 2024 Aug 16;10(8):583. doi: 10.3390/jof10080583 (PMC11355219; doi:10.3390/jof10080583)

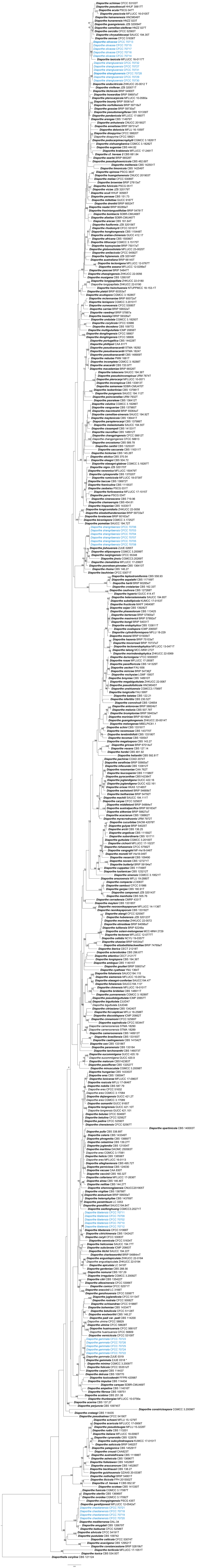

Supplement: Supplementary file 1 [file Figure S5.pdf › Supplementary Files/Figure S5.pdf]
